# Hydrodynamic Radii of Intrinsically Disordered Proteins Determined from Experimental Polyproline II Propensities

**DOI:** 10.1371/journal.pcbi.1004686

**Published:** 2016-01-04

**Authors:** Maria E. Tomasso, Micheal J. Tarver, Deepa Devarajan, Steven T. Whitten

**Affiliations:** Department of Chemistry and Biochemistry, Texas State University, San Marcos, Texas, United States of America; Indiana University, UNITED STATES

## Abstract

The properties of disordered proteins are thought to depend on intrinsic conformational propensities for polyproline II (*PP*_*II*_) structure. While intrinsic *PP*_*II*_ propensities have been measured for the common biological amino acids in short peptides, the ability of these experimentally determined propensities to quantitatively reproduce structural behavior in intrinsically disordered proteins (IDPs) has not been established. Presented here are results from molecular simulations of disordered proteins showing that the hydrodynamic radius (*R*_*h*_) can be predicted from experimental *PP*_*II*_ propensities with good agreement, even when charge-based considerations are omitted. The simulations demonstrate that *R*_*h*_ and chain propensity for *PP*_*II*_ structure are linked via a simple power-law scaling relationship, which was tested using the experimental *R*_*h*_ of 22 IDPs covering a wide range of peptide lengths, net charge, and sequence composition. Charge effects on *R*_*h*_ were found to be generally weak when compared to *PP*_*II*_ effects on *R*_*h*_. Results from this study indicate that the hydrodynamic dimensions of IDPs are evidence of considerable sequence-dependent backbone propensities for *PP*_*II*_ structure that qualitatively, if not quantitatively, match conformational propensities measured in peptides.

## Author Summary

Molecular models of disordered protein structures are needed to elucidate the functional mechanisms of intrinsically disordered proteins, a class of proteins implicated in many disease pathologies and human health issues. Several studies have measured intrinsic conformational propensities for polyproline II helix, a key structural motif of disordered proteins, in short peptides. Whether or not these experimental polyproline II propensities, which vary by amino acid type, reproduce structural behavior in intrinsically disordered proteins has yet to be demonstrated. Presented here are simulation results showing that polyproline II propensities from short peptides accurately describe sequence-dependent variability in the hydrodynamic dimensions of intrinsically disordered proteins. Good agreement was observed from a simple molecular model even when charge-based considerations were ignored, predicting that global organization of disordered protein structure is strongly dependent on intrinsic conformational propensities and, for many intrinsically disordered proteins, modulated only weakly by coulombic effects.

## Introduction

Many proteins, and protein domains, that perform critical biological tasks have disordered structures under normal solution conditions [1–3]. These proteins are referred to as intrinsically disordered [4] and, accordingly, molecular models of disordered protein structures are needed to understand the physical basis for the activities [2,3], roles regulating key signaling pathways [5], and relationships to human health issues [6–9] that have been linked to intrinsically disordered proteins (IDPs).

The properties of disordered protein structures are often associated with conformational propensities for polyproline II (*PP*_*II*_) helix [10–12] and charge-based intramolecular interactions [13–15]. *PP*_*II*_ propensities are locally-determined [16] and intrinsic to amino acid type [17–19], while charge-charge interactions seem to be important for organizing disordered structures owing to both long and short range contacts [13–15,20,21]. Since chain preferences for *PP*_*II*_ increase the hydrodynamic sizes of IDPs [22,23], and Coulombic interaction energies are distance-dependent, it could be argued that charge effects on IDP structures are modulated locally by intrinsic *PP*_*II*_ propensities. A number of issues with that hypothesis, however, are apparent. First, it has not been established if *PP*_*II*_ propensities measured in short peptide models of the unfolded states of proteins [17–19] translate to IDPs. It could be that *PP*_*II*_ propensities are negligible and unimportant in IDP systems. Second, methods capable of separating the impact of weak to possibly strong local conformational propensities and charge-charge interactions in the context of flexible and disordered protein structures have not been demonstrated, but are required for testing any potential interdependence.

To investigate such issues, a computer algorithm [22–24] based on the Hard Sphere Collision (HSC) model [25] was developed for parsing the contributions of intrinsic *PP*_*II*_ propensities and charge to the structures of IDPs, as represented by the hydrodynamic radius (*R*_*h*_). A HSC model was chosen since *PP*_*II*_ propensities and charge effects could be added separately and in steps, to isolate contributions to simulated IDP structures. *R*_*h*_ was chosen since experimental values are available for a wide range of IDP sequences, allowing direct comparisons to model-simulated *R*_*h*_.

Here we demonstrate that *R*_*h*_ for disordered proteins trend with chain propensities for *PP*_*II*_ structure by a simple power-law scaling relationship. Using experimental *PP*_*II*_ propensities for the common biological amino acids from Kallenbach [17], Creamer [18], and Hilser [19], this relationship was tested against experimental *R*_*h*_ from 22 IDPs [23,26–42] ranging in size from 73 to 260 residues and net charge from 1 to 43. We observed that the power-law scaling function was able to reproduce IDP *R*_*h*_ with good agreement when using propensities from Hilser, while the Kallenbach and Creamer scales consistently overestimated *R*_*h*_. The ability to describe *R*_*h*_ from just intrinsic *PP*_*II*_ propensities associated with a sequence was supported by simulation results showing that charge effects on IDP *R*_*h*_ are generally weak. Relative to the effects of *PP*_*II*_ propensities, charge effects on IDP *R*_*h*_ were substantial only when charged side chains were separated in sequence by 2 or fewer residue positions and if the sequence had higher than typical bias for one charge type (i.e., positive or negative). Overall, these results demonstrated that two seemingly disparate experimental datasets, IDP *R*_*h*_ and intrinsic *PP*_*II*_ propensities, are in qualitative agreement; providing evidence for considerable sequence-dependent conformational preferences for *PP*_*II*_ structure in the disordered states of biological proteins.

## Results

### Computer simulation of *R*_*h*_ dependence on *PP*_*II*_ propensity

**Fig 1:**
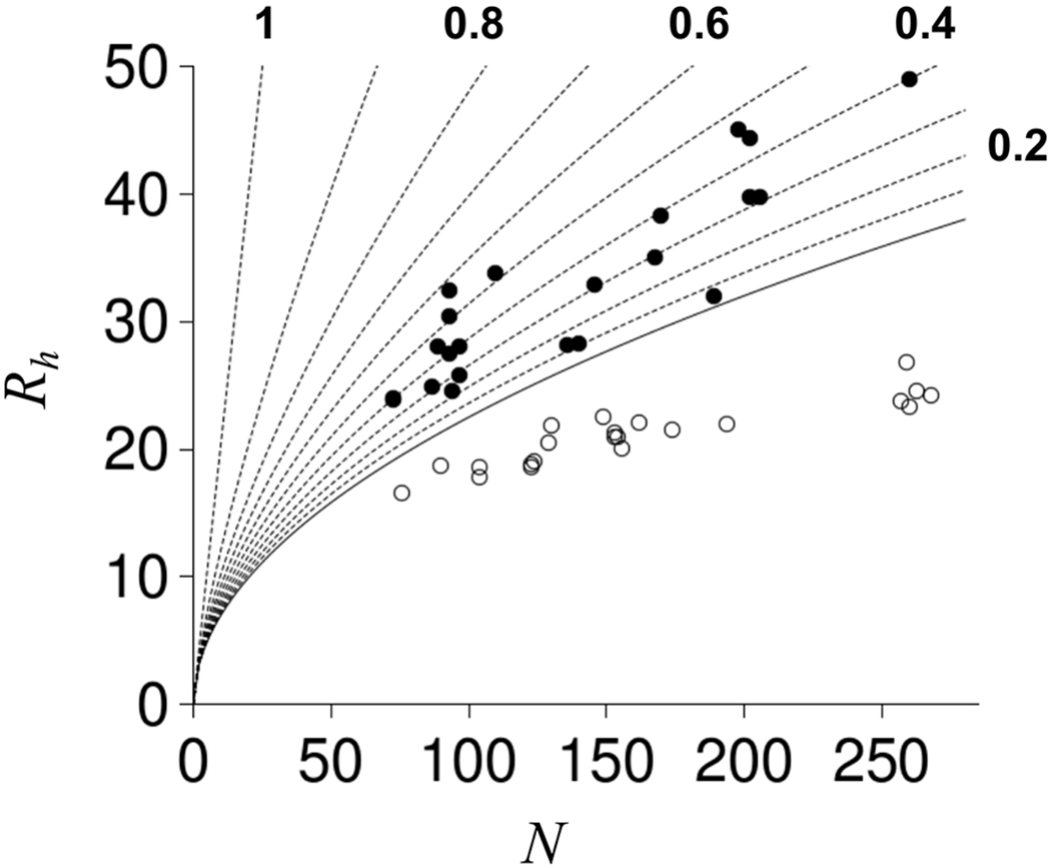
*R*_*h*_ comparison to number of residues, *N*. Filled and open circles represent experimental *R*_*h*_ for IDPs [23,26–42] and folded proteins [22,45,46], respectively. The solid line is the *R*_*h*_ dependence on *N* estimated from simulations of randomly configured protein structures [22]. Stippled lines show *R*_*h*_ for randomly configured structures with chain propensities for *PP*_*II*_ (*f*_*PPII*_) from 0.1 to 1 in 0.1 increments. Every other stippled line is end-labeled by its *f*_*PPII*_ value.

*R*_*h*_ for IDPs are sensitive to site-specific and general structural perturbations such as amino acid substitutions [23], changes in net charge [13,14], charge rearrangements [15], and temperature changes [22,43,44]. Fig 1 shows that IDP *R*_*h*_ differ substantially from *R*_*h*_ for folded proteins [22,45,46] that have similar residue length, *N*. *R*_*h*_ from modeling proteins with no strongly preferred conformations [22], which is referred to as a random coil [47], is also provided for comparison to the experimental values. The solid line representing coil *R*_*h*_ was determined from computer simulation of randomly configured polypeptide chains using a HSC model [22]. Owing to favorable native contacts that promote stable globular structures, folded proteins have *R*_*h*_ that are compacted relative to the *R*_*h*_ of simulated random coils. In contrast, the data in Fig 1 indicate that *R*_*h*_ from IDPs are generally larger than random coil estimates.

The dependence of *R*_*h*_ on *N* for chemically denatured proteins follows a power-law scaling relationship,

$$R_{h}=R_{o}\cdot N^{v},$$ (1)

where *R*_*o*_ is 2.2 Å and *v* is 0.57 [45]. To understand changes in *R*_*o*_ and *v* that are required for modeling the dependence of *R*_*h*_ on *N* for IDPs, it is useful to recognize that unfolded proteins in aqueous solutions absent high concentrations of guanidine hydrochloride or urea show *R*_*h*_ compaction [48] with a concomitant decrease in *v* [49]. Consistent with that observation, Marsh and Forman-Kay demonstrated that *R*_*h*_ and *N* scale with *v* = 0.509 for IDPs under normal conditions [49]. *R*_*o*_ for IDPs was found to depend on PRO content and net charge by,

$$R_{o}=\left(1.24\cdot f_{PRO}+0.904\right)\cdot \left(0.00759\cdot \left|Q\right|+0.963\right)\cdot 2.49,$$ (2)

where *f*_*PRO*_ is the fractional number of PRO residues and |*Q*| the absolute net charge determined from sequence [49]. Since PRO residues have strong propensities for *PP*_*II*_ helix, which is an extended structure [50], and repulsive interactions between charged groups likewise favor extended conformations to minimize unfavorable energetics, a simple molecular interpretation of Eq (2) can be offered whereby the *R*_*h*_ dependence on *N* for IDPs follows a baseline trend of *R*_*h*_ = (2.17 Å)∙*N* ^0.509^ (i.e., *R*_*o*_ with *f*_*PRO*_ and |*Q*| set to zero) with sequence-dependent increases in *R*_*h*_ from this baseline owing to chain propensities for *PP*_*II*_ and repulsive charge-charge interactions. Simulated *R*_*h*_ for random coils were observed to trend with *N* by *R*_*h*_ = (2.16 Å)∙*N* ^0.509^ [22], supporting this hypothesis (and reproduced in Fig 1). The effects of ALA to GLY substitutions on IDP *R*_*h*_ also indicated that chain propensities for *PP*_*II*_ structure modulate IDP *R*_*h*_ and not simply PRO content [23].

To model the effects of *PP*_*II*_ propensities on coil *R*_*h*_, a sampling bias for *PP*_*II*_ structure was applied to random coil simulations and the relationship between *R*_*h*_, *N*, and fractional number of residues in the *PP*_*II*_ conformation, *f*_*PPII*_, was determined [22,23]. This is shown in Fig 1 by stippled lines to demonstrate that increases in *f*_*PPII*_ cause increases in coil *R*_*h*_. These results were generated from simulations that modeled *PP*_*II*_ bias by applying an identical sampling bias for *PP*_*II*_ structure at each residue position in a polypeptide chain and, accordingly, did not include effects that could be caused by position-specific variations in *PP*_*II*_ propensity.

To test for effects on coil *R*_*h*_ owing to *PP*_*II*_ propensity variations within a polypeptide chain, conformational ensembles for *N* = 15, 25, 35, 50, and 75 were generated for poly-ALA with the algorithm modified to allow position-specific sampling rates for *PP*_*II*_ structure. It was shown previously that the effects of *N* on *R*_*h*_ were mostly insensitive to amino acid sequence in HSC model simulations of disordered proteins [22] and thus poly-ALA was chosen as a computational simplification. Variations in *PP*_*II*_ propensity among residue positions were simulated by applying a sampling bias for *PP*_*II*_ structure (*S*_*PPII*_) at every position, every second position, every third position, every fourth position, or every fifth position in the poly-ALA chains. *S*_*PPII*_ at values of 0.1, 0.2, 0.3, 0.4, 0.5, 0.6, 0.7, 0.8, and 0.9 were tested at the indicated residue locations. This *PP*_*II*_ sampling strategy resulted in 225 separate simulated ensembles (5 *N* lengths X 5 patterns X 9 *S*_*PPII*_ values).

A set of simulations using randomly determined position-specific bias for *PP*_*II*_ structure was also modeled using poly-ALA chains. These additional simulations used *N* = 15, 25, and 35, with each residue position assigned a different random value for *S*_*PPII*_. Position-specific random assignments were repeated 3 times for *S*_*PPII*_ ranging from 0 to 1, 0 to 0.5, 0.25 to 0.75, and 0.5 to 1, resulting in an additional 36 simulated ensembles (3 *N* lengths X 3 distributions of random position-specific *PP*_*II*_ biases X 4 applied ranges in *PP*_*II*_ sampling bias).

The ensemble-averaged fractional number of residues in the *PP*_*II*_ conformation (i.e., the propensity) can be different from *S*_*PPII*_ in these simulations since randomly generated structures containing van der Waals contact violations are removed from the calculation. Differences between the applied sampling rate (i.e., *S*_*PPII*_) and the observed ensemble-averaged rate (i.e., *f*_*PPII*_) at *S*_*PPII*_-targeted positions followed the same Gaussian relationship that was established previously for whole-chain *S*_*PPII*_ and *f*_*PPII*_ comparisons [22] and thus straight-forward conversion between applied and observed bias rates was available (S1 Fig). *f*_*PPII*_ determined from simulation for residue positions with no applied *S*_*PPII*_ was 0.012 ± 0.004.

**Fig 2:**
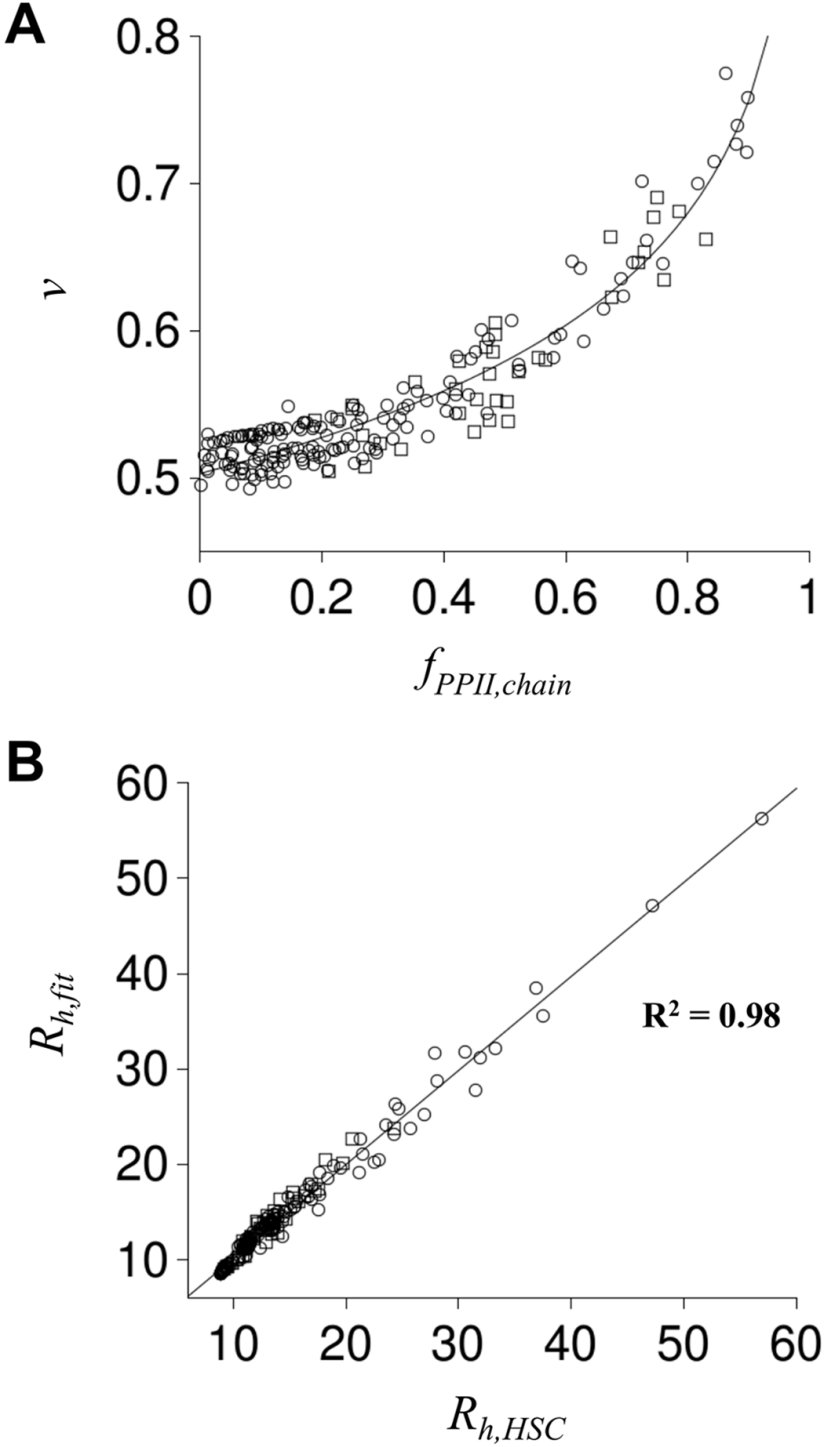
Simulated effect of *PP*_*II*_ propensities on coil *R*_*h*_. Each circle and square represents a simulated disordered polypeptide. Squares are from ensembles simulated with position-specific *PP*_*II*_ propensities assigned randomly; circles had *PP*_*II*_ propensity assignments that followed the sequence patterns described in the text. In panel **A**, *f*_*PPII*,*chain*_ was calculated as <*N*_*PPII*_>/*N*, where <*N*_*PPII*_> was the ensemble averaged number of residues with (Φ,Ψ) in the *PP*_*II*_ region (-75±10, 145±10), and *v* was calculated as ln(*R*_*h*_/*R*_*o*_)/ln(*N*) using <*L*>/2 for *R*_*h*_ and 2.16 Å for *R*_*o*_. These data were fit to *v* = *v*_*o*_ + *β*∙ln(1-*f*_*PPII*,*chain*_), with *v*_*o*_ and *β* as fit parameters, producing the solid line. In panel **B**, *R*_*h*,*HSC*_ was calculated as <*L*>/2. *R*_*h*,*fit*_ was determined from *f*_*PPII*,*chain*_ using *R*_*h*,*fit*_ = (2.16 Å)∙*N* ^*v*^ and the panel A fit for *v*. *R*_*h*,*HSC*_ and *R*_*h*,*fit*_ correlation (R^2^) is provided in the figure.

Cumulative results from the >250 separate ensemble simulations were analyzed in terms of the power-law scaling relationship given by Eq (1). Previously, we demonstrated that the exponential term, *v*, was dependent on *S*_*PPII*_ while *R*_*o*_ was mostly independent of *S*_*PPII*_ with an averaged value of 2.16 Å [22]. Fig 2A shows *v*, determined from ln(*R*_*h*_/2.16)/ln(*N*), for each simulated ensemble and plotted as a function of *f*_*PPII*_ calculated for the whole chain. *R*_*h*_ for each simulated ensemble was calculated as,

$$R_{h}=\langle L\rangle /2,$$ (3)

and *f*_*PPII*,*chain*_ as,

$$f_{PPII,chain}=\langle N_{PPII}\rangle /N\cdot$$ (4)

In Eq (3), <*L*> = ∑ *L*_*i*_∙*P*_*i*_, where *L*_*i*_ is the maximum Cα-Cα distance calculated for state *i*, *P*_*i*_ is the Boltzmann probability for state *i*, and the summation was over all states *i* of an ensemble. In Eq (4), <*N*_*PPII*_> = ∑ *N*_*PPII*,*i*_∙*P*_*i*_, where *N*_*PPII*,*i*_ is the number of residues in the *PP*_*II*_ conformation for state *i*. The distinction of “chain” given to *f*_*PPII*_ in Eq (4) was provided to limit confusion between *f*_*PPII*_ calculated for a whole chain versus *f*_*PPII*_ calculated for specific residue positions.

The relationship between *v* and *f*_*PPII*,*chain*_ for all simulations followed a logarithmic trend that was fit to the equation,

$$v\left(f_{PPII,chain}\right)=v_{o}+\beta \cdot ln\left(1-f_{PPII,chain}\right),$$ (5)

using the Levenberg-Marquardt method of nonlinear least squares [51,52]. The parameters *v*_*o*_ and *β* were found to be 0.503 ± 0.002 and -0.11 ± 0.003, respectively. Fig 2B shows that *R*_*h*_ determined from *f*_*PPII*,*chain*_ (Eq (4)) and *N* by combining Eqs (1) and (5) (see Eq (6) below) correlated strongly with *R*_*h*_ calculated directly from a simulated ensemble (Eq (3)). All possible patterns of position-specific *PP*_*II*_ bias were not tested in our computer trials. Results in Fig 2 predict, however, that in general a quantitative relationship exists for disordered proteins between *R*_*h*_, *N*, and the ensemble-averaged per-residue chain propensity for *PP*_*II*_ structure (*f*_*PPII*,*chain*_).

### Test of model using experimental *PP*_*II*_ propensities

Results from HSC model simulations that are summarized in Figs 1 and 2 can be interpreted as an ideal relationship between *R*_*h*_ and *N* that includes the general effects of sterics and *PP*_*II*_ propensities but is absent other intrinsic and intramolecular factors. Contributions from Coulombic interaction energies to IDP *R*_*h*_ will be discussed below and added to this model. First, the simulation-derived relationship between *R*_*h*_, *N*, and *f*_*PPII*,*chain*_ is tested by applying experimental *PP*_*II*_ propensities to the sequences of IDPs in Fig 1. The identity, sequence, and experimental *R*_*h*_ for each IDP are given in Supporting Information (S1 and S2 Tables). This dataset includes 22 IDPs containing 3016 total residue positions. Amino acids represented at rates greater than 0.05 in this dataset were, in rank order and listed by their three letter codes, SER (0.104), GLU (0.100), LEU (0.083), PRO (0.080), ASP (0.074), GLY (0.073), ALA (0.073), THR (0.061), LYS (0.055), GLN (0.053), and VAL (0.053).

Amino acid *PP*_*II*_ propensities reported by Kallenbach [17], Creamer [18], and Hilser [19] for disordered proteins are reproduced in Table 1 and were used for testing the relationship,

$$R_{h}=2.16\cdot N^{0.503-0.11\cdot ln\left(1-f_{PPII,chain}\right)}\cdot$$ (6)

**Table 1:** Intrinsic backbone *PP*_*II*_ propensities measured in disordered peptides.

	Kallenbach [17]	Creamer [18]	Hilser [19]
*host* ^a^	Ac-G_2_XG_2_-NH_2_	Ac-P_3_XP_3_GY-NH_2_	Ac-VP_2_XVP_2_R_3_Y-NH_2_
ALA (A)	0.818	0.61	0.37
CYS (C)	0.557	0.55	0.25
ASP (D)	0.552	0.63	0.30
GLU (E)	0.684	0.61	0.42
PHE (F)	0.639	0.58	0.17
GLY (G)	-	0.58	0.13
HIS (H)	0.428	0.55	0.20
ILE (I)	0.519	0.50	0.39
LYS (K)	0.581	0.59	0.56
LEU (L)	0.574	0.58	0.24
MET (M)	0.498	0.55	0.36
ASN (N)	0.667	0.55	0.27
PRO (P)	-	0.67	1.00
GLN (Q)	0.654	0.66	0.53
ARG (R)	0.638	0.61	0.38
SER (S)	0.774	0.58	0.24
THR (T)	0.553	0.53	0.32
VAL (V)	0.743	0.49	0.39
TRP (W)	0.764	-	0.25
TYR (Y)	0.630	-	0.25
*average*	0.626	0.58	0.35

^*a*^sequence of host peptide used to measure *PP*_*II*_ propensity at the guest position, X

These propensity scales were chosen since weak correlations are observed among the group (S2 Fig), indicating a potential for yielding different results when each set is used separately with Eq (6) for a given IDP sequence. A physical explanation for the different *PP*_*II*_ propensity values reported for the amino acids is not given here (e.g., the reported ALA *PP*_*II*_ propensities are very different when compared), other than to note that their measurements used host peptide sequences that were also very different (Table 1). Kallenbach measured *PP*_*II*_ propensities in the background of a GLY-rich host peptide, whereas the scale reported by Creamer was determined for positions flanked on both sides by PRO residues. The propensity scale from Hilser was measured for positions located in between PRO and valine (VAL). Other *PP*_*II*_ propensity scales were not included in these tests due to similarities to the Kallenbach, Creamer, or Hilser reported values. For example, a *PP*_*II*_ propensity scale from Zondlo [53] correlated with the Creamer values (coefficient of determination, R^2^, gave a correlation of 0.58), likely owing to the use of a host peptide that also flanked the guest position with PRO residues.

Inspection of Table 1 shows that *PP*_*II*_ propensities for tryptophan (TRP) and tyrosine (TYR) were not reported by Creamer. For these amino acids, we used the averaged Creamer-reported value calculated from the 18 other amino acids (0.58). In the Hilser set, TRP and TYR had lower than average *PP*_*II*_ propensity. In contrast, TRP and TYR had higher than average *PP*_*II*_ propensity in the Kallenbach set. Using the Creamer average was a compromise that likely had low significance in our tests since TRP and TYR had very low representation among the IDP sequences; 0.008 and 0.012, respectively. *PP*_*II*_ propensities were not reported for PRO and GLY by Kallenbach. Here, we used 1 for PRO since it is generally accepted that PRO has the highest propensity for *PP*_*II*_ structure [10,12,17–19]. This gave PRO a larger value than ALA (0.818), which was the amino acid with the highest reported propensity in the Kallenbach set. GLY was given a propensity of 0.50, which is lower than the Kallenbach average (0.626) but higher than the lowest value (0.428). This also was a compromise from observing that GLY had the lowest value in the Hilser set (0.13), but an average value in the Creamer set (0.58).

**Fig 3:**
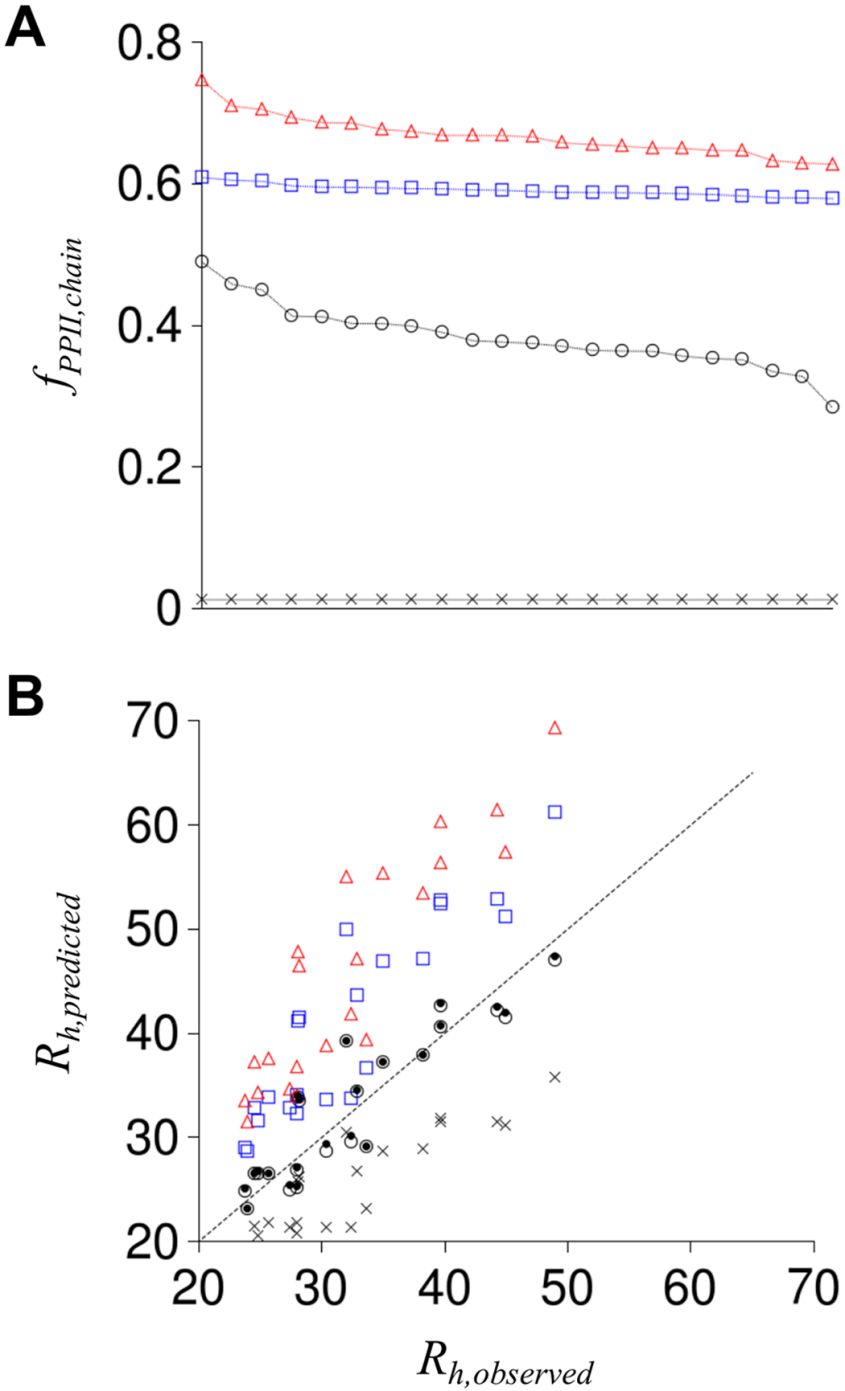
Chain propensity for *PP*_*II*_ from experimental scales and comparison of predicted and observed *R*_*h*_. Panel **A** gives *f*_*PPII*,*chain*_ for each IDP sequence, ordered left to right to show the range obtained with each scale, calculated using experimental *PP*_*II*_ propensities from Kallenbach (red triangles), Creamer (blue squares), and Hilser (open circles). X is *f*_*PPII*,*chain*_ from the null model. Panel **B** shows *R*_*h*_ predicted for each IDP using Eq (6) and *f*_*PPII*,*chain*_ from panel A. Symbols in panel B match panel A representations. Black dots show *R*_*h*_ predicted from the composite propensity scale. Stippled line is the identity line.

*f*_*PPII*,*chain*_ was calculated for each IDP by using the amino acid *PP*_*II*_ propensity given in Table 1, summing over the IDP sequence, and dividing by *N*. Fig 3A shows the experimental scales predict different chain propensities for *PP*_*II*_ structure for each IDP sequence. The scale from Kallenbach gave *f*_*PPII*,*chain*_ ranging from 0.746 to 0.628, whereas the Creamer and Hilser scales gave *f*_*PPII*,*chain*_ from 0.609 to 0.579 and 0.489 to 0.283, respectively. Eq (6) was then used to predict *R*_*h*_ from *f*_*PPII*,*chain*_ for comparison to experimentally observed *R*_*h*_, which is shown in Fig 3B. The average prediction error (|*R*_*h*,*predicted*_−*R*_*h*,*observed*_|) and the correlation between predicted and observed *R*_*h*_ is given in Table 2. To assess contributions from the amino acid scales for predicting *R*_*h*_, a null model was included by assigning each amino acid the *PP*_*II*_ propensity of 0.012, the background *f*_*PPII*_ calculated from HSC simulations when no sampling bias for *PP*_*II*_ structure was applied (i.e., *S*_*PPII*_ = 0). Accordingly, the null model represents random coil values.

**Table 2:** Comparison of predicted and observed *R*_*h*_.

Propensity Scale	Average Error (Å)^a^	R^2^ ^b^	Average Normalized Error^c^	R^2^ ^d^
Null (random coil)	7.1 ± 3.7	0.797	-0.28 ± 0.13	0.265
Kallenbach	13.4 ± 5.4	0.819	0.51 ± 0.15	0.301
Creamer	8.4 ± 4.3	0.817	0.32 ± 0.13	0.297
Hilser	2.5 ± 1.8	0.825	0.006 ± 0.12	0.407
Composite	2.4 ± 1.8	0.834	0.015 ± 0.12	0.423
Static	2.6 ± 2.0	0.799	-0.016 ± 0.13	0.291

^*a*^determined from |predicted *R*_*h*_—observed *R*_*h*_|

^*b*^coefficient of determination, correlation of predicted *R*_*h*_ and observed *R*_*h*_

^*c*^determined from (predicted *R*_*h*_—observed *R*_*h*_)/(random coil *R*_*h*_)

^*d*^coefficient of determination, correlation of normalized error and net charge density

Different values of *f*_*PPII*,*chain*_ predict different *R*_*h*_ for a given IDP sequence, as expected from Eq (6). For example, the null model, which used the smallest *f*_*PPII*,*chain*_ values, predict *R*_*h*_ that are smaller than observed for each IDP. In contrast, *PP*_*II*_ propensities from Kallenbach and Creamer, which report relatively large *f*_*PPII*,*chain*_ values, predict *R*_*h*_ that are larger than observed for each IDP. Experimental propensities from Hilser predict *R*_*h*_ that trend with the identity line, showing good agreement, but also showing scatter relative to that line (average error was 2.5 Å). In an attempt to reduce prediction error, a composite *PP*_*II*_ propensity scale that used the Hilser values by default but the Kallenbach values for residues located between GLY (i.e., GLY-X-GLY) and Creamer values for residues located between PRO (i.e., PRO-X-PRO) was tested. This context-specific composite propensity scale (identified as “Composite” in Table 2 and Fig 3B) caused only small changes in predicted *R*_*h*_, with no significant improvement in prediction capabilities relative to using only the Hilser reported *PP*_*II*_ propensities.

Since *R*_*h*_ increases with *N* (Fig 1), prediction error was normalized for peptide length by,

$$\text{normalized error }=\;(\text{predicted }R_{h}\;-\text{ observed }R_{h})/(\text{random coil }R_{h})\cdot$$ (7)

**Fig 4:**
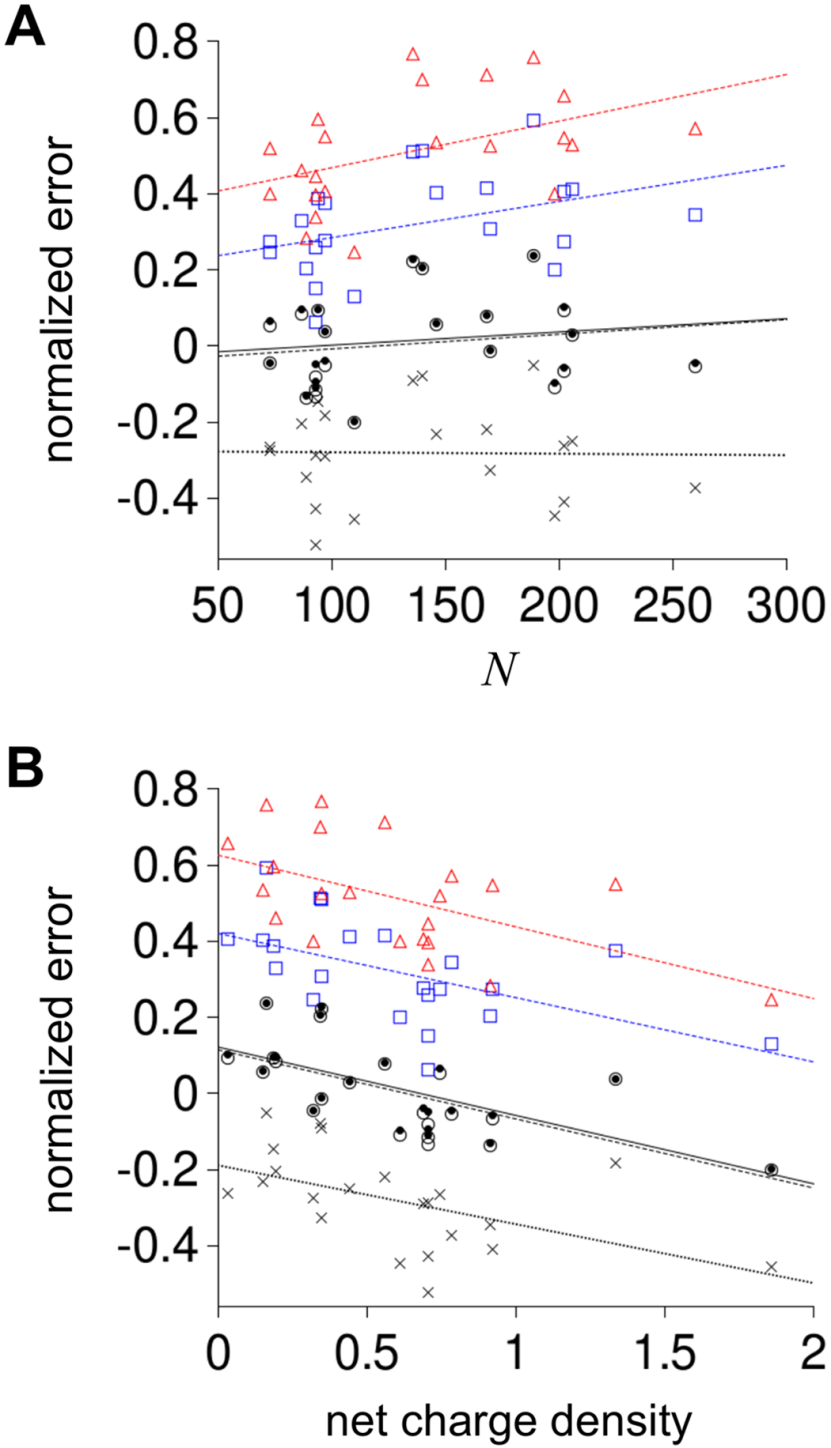
Correlation of normalized error in predicted *R*_*h*_ to *N* and net charge density. Normalized error and net charge density were calculated for each IDP using Eqs (7) and (8), respectively. In both panels, red triangles show normalized error from *R*_*h*_ predicted using the Kallenbach reported propensities, blue squares from Creamer reported propensities, open circles from Hilser reported propensities, black dots from the composite propensity scale, and X is the null model. Lines are linear fits to the five prediction sets colored as the symbols (Kallenbach scale was red; Creamer was blue, Hilser was stippled black, composite was solid black, and null was dotted black).

Random coil *R*_*h*_ was calculated using Eq (6) with *f*_*PPII*,*chain*_ = 0.012, the null model value. Average normalized error is given in Table 2 for each propensity scale. Fig 4 shows trends in the normalized error with *N* and net charge density, determined as the absolute net charge normalized for peptide length,

$$\text{net charge density }=\;\left|Q\right|/(\text{random coil }R_{h})\cdot$$ (8)

S1 Table gives net charge and *N* for each IDP. No obvious bias with peptide length (i.e., *N*) was observed in the normalized error for the Hilser and composite propensity scales. Normalized error clearly increased with *N* when using Kallenbach and Creamer values, indicating that these *PP*_*II*_ propensities may be over-estimated when applied to IDP sequences to predict *R*_*h*_. Since the exponent in Eq (6) becomes larger with increasing *f*_*PPII*,*chain*_, a set of propensity values that systematically are too large would cause normalized errors that increase with *N*.

It is interesting to note that normalized error correlated with net charge density for each experimental propensity scale (Fig 4B and Table 2), suggesting that prediction error was caused partially by charge effects on *R*_*h*_ that were not included in the model. This is not surprising since Marsh and Forman-Kay demonstrated that increases in net charge correlate with increases in IDP *R*_*h*_ [49] and the trend we observed of decreasing normalized error with increased net charge density is consistent with their conclusions. Extrapolating this trend to zero net charge density for the Hilser and composite propensity scales yields positive normalized errors suggesting that, in the background of no net charge contributions to *R*_*h*_, the *PP*_*II*_ propensities reported by Hilser may also be slightly too large when using Eq (6) to predict *R*_*h*_.

While this analysis of experimental *PP*_*II*_ propensities indicated that one of the scales was capable of reproducing experimental *R*_*h*_ with good agreement for a set of IDPs, it is important to recognize that comparative tests based on Eq (6) may not be suitable for affirmation. Since *R*_*h*_ in this model depends only on *N* and chain averaged propensity for *PP*_*II*_ structure, contrived scales that predict IDP *R*_*h*_ with similar agreement in terms of the average prediction error are simple to generate. For example, each IDP could be given a sequence-independent *f*_*PPII*,*chain*_ value of 0.364, which was determined by converting experimental *R*_*h*_ to an apparent *f*_*PPII*,*chain*_ using Eq (6) and then averaging over the IDP dataset. Using this static *f*_*PPII*,*chain*_ to predict IDP *R*_*h*_ gives an average prediction error (identified as “Static” in Table 2) that is close to the error obtained when using the experimental scale from Hilser. Correlations between predicted and observed *R*_*h*_ and between normalized error and net charge density for the contrived static scale, however, decreased relative to the correlations that were observed with the experimental scales, suggesting that static representations of *f*_*PPII*,*chain*_ may not fully capture some molecular dependencies that are inherent to IDP *R*_*h*_.

**Fig 5:**
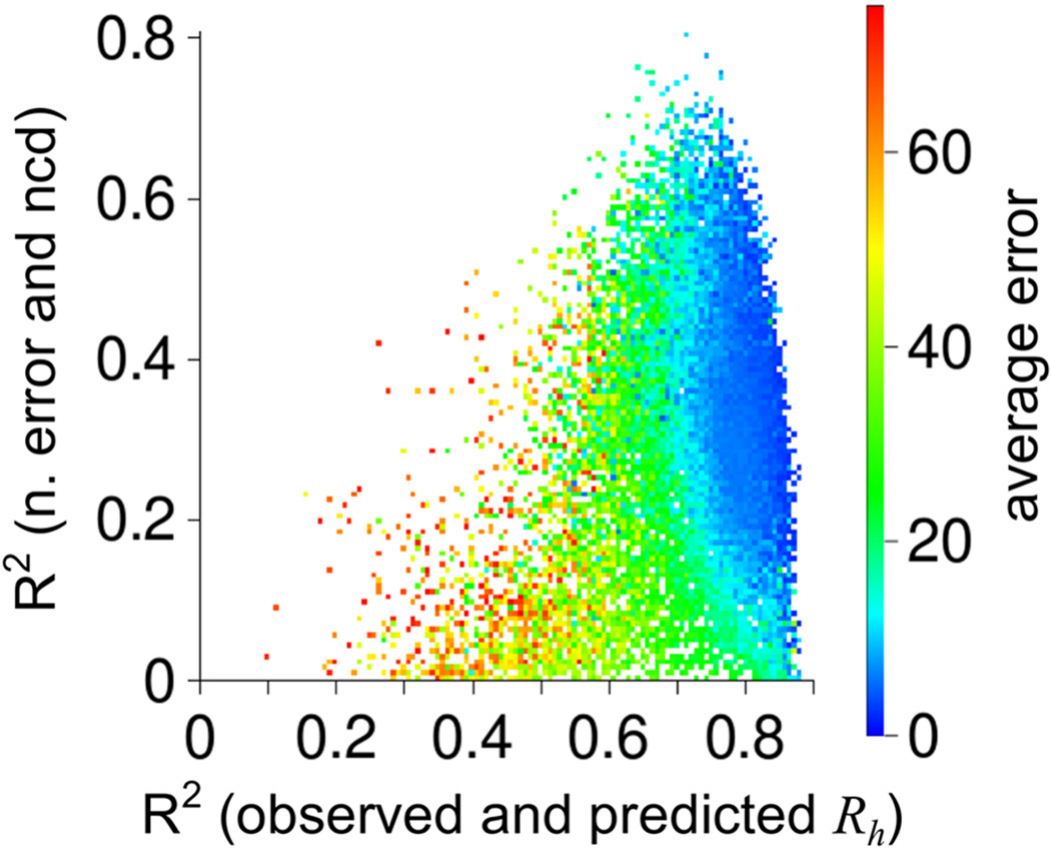
*R*_*h*_ prediction from random *PP*_*II*_ propensity scales. Random scales were generated as described in the text and used to predict *R*_*h*_ for each IDP by Eq (6). Shown is the correlation (R^2^) obtained for each scale between observed and predicted *R*_*h*_ plotted against the correlation obtained between the normalized error (n. error) and the net charge density (ncd). Shown by color is the average prediction error of each scale. Random scales giving average prediction error larger than 75 Å were omitted to emphasize differences at lower error values.

To further investigate the capabilities of Eq (6) for relating IDP *R*_*h*_ and *PP*_*II*_ propensity, random sets of amino acid scales were generated following a two-step protocol and analyzed. First, a random number between 0 and 1 was used to target an average propensity for a scale. Then, random scales were generated, where each amino acid was assigned a different random value between 0 and 1, until a set was found whose average for the 20 amino acids matched the target determined in the first step (±0.05). The goal from using two steps to generate scales was to ensure that chain averaged propensities in the high, medium, and low range were evenly sampled. This sampling scheme was repeated until 100,000 random scales were generated. Each propensity scale was then used to predict *R*_*h*_ from Eq (6) and the results are summarized in Fig 5. It was observed that randomly generated scales gave average prediction errors for the IDP dataset ranging from 1.9 to 239.8 Å, correlations between predicted and observed *R*_*h*_ ranging from 0.02 to 0.88, and correlations between normalized error and net charge density from 0 to 0.81. Optimal values for these metrics (i.e., highest correlations coupled with lowest average error), seem to focus toward values of R^2^ and average error that are obtained when using experimental *PP*_*II*_ propensities from Hilser. This result shows that experimental *R*_*h*_ of the IDP dataset are in good qualitative agreement with experimental *PP*_*II*_ propensities reported by Hilser, and vice versa, giving evidence that the molecular properties of IDPs that link *R*_*h*_, *N*, and *f*_*PPII*,*chain*_ are well-approximated by the simple power-law scaling relationship of Eq (6).

### Effects of Coulombic interaction energies on *R*_*h*_

In the HSC model used for this study, a computer algorithm generates polypeptide structures by random conformational search until *R*_*h*_ (Eq (3)) converges to a stable ensemble-averaged value [22]. A structure-based energy function parameterized to solvent-accessible surface areas that has been tested extensively [54–62] is used to population-weight each randomly generated structure. To approximate charge effects on ensemble populations, the energy function was modified to include Coulombic interaction energies by,

$$\Delta G_{Coulomb}=\frac{332}{D_{H2O}\cdot 2}\cdot \sum_{i}Z_{i}\cdot \left(\sum_{j}\frac{Z_{j}}{R_{ij}\cdot e^{\kappa \cdot R_{ij}}}\right),$$ (9)

where the constant 332 converts the energy into units of kilocalories per mole at 25°C, *D*_*H2O*_ is the dielectric of water, *Z* is the charge at site *i* or *j*, *R*_*ij*_ is the distance between two charged sites *i* and *j* (in Å), κ (the Debye parameter) accounts for screening from solution ionic strength, and the sums are over all charge-bearing sites. The Debye parameter was calculated as,

$$\kappa =2.913\cdot \sqrt{I/D_{H2O}},$$ (10)

where *I* is ionic strength (in molarity, *M*). *D*_*H2O*_ used was 78.3 [63] and *I* was 0.1 *M* to represent normal conditions. Since the simulations used poly-ALA chains, charged residues were modeled with a positive or negative charge located at the coordinates of the Cβ atom to denote the approximate location for flexible and charged side chains. Coordinates for the backbone N and O atoms of the first and last residues were used to assign positive and negative charge, respectively, to N- and C-termini. Simulations were limited to 25 residue poly-ALA chains to establish trends for the effects of charge on *R*_*h*_ in this model. For each ensemble, an identical *S*_*PPII*_ was applied at each residue position. *S*_*PPII*_ was varied among the different simulations to target ensemble-averaged *f*_*PPII*,*chain*_ ranging from 0.1 to 0.92.

**Fig 6:**
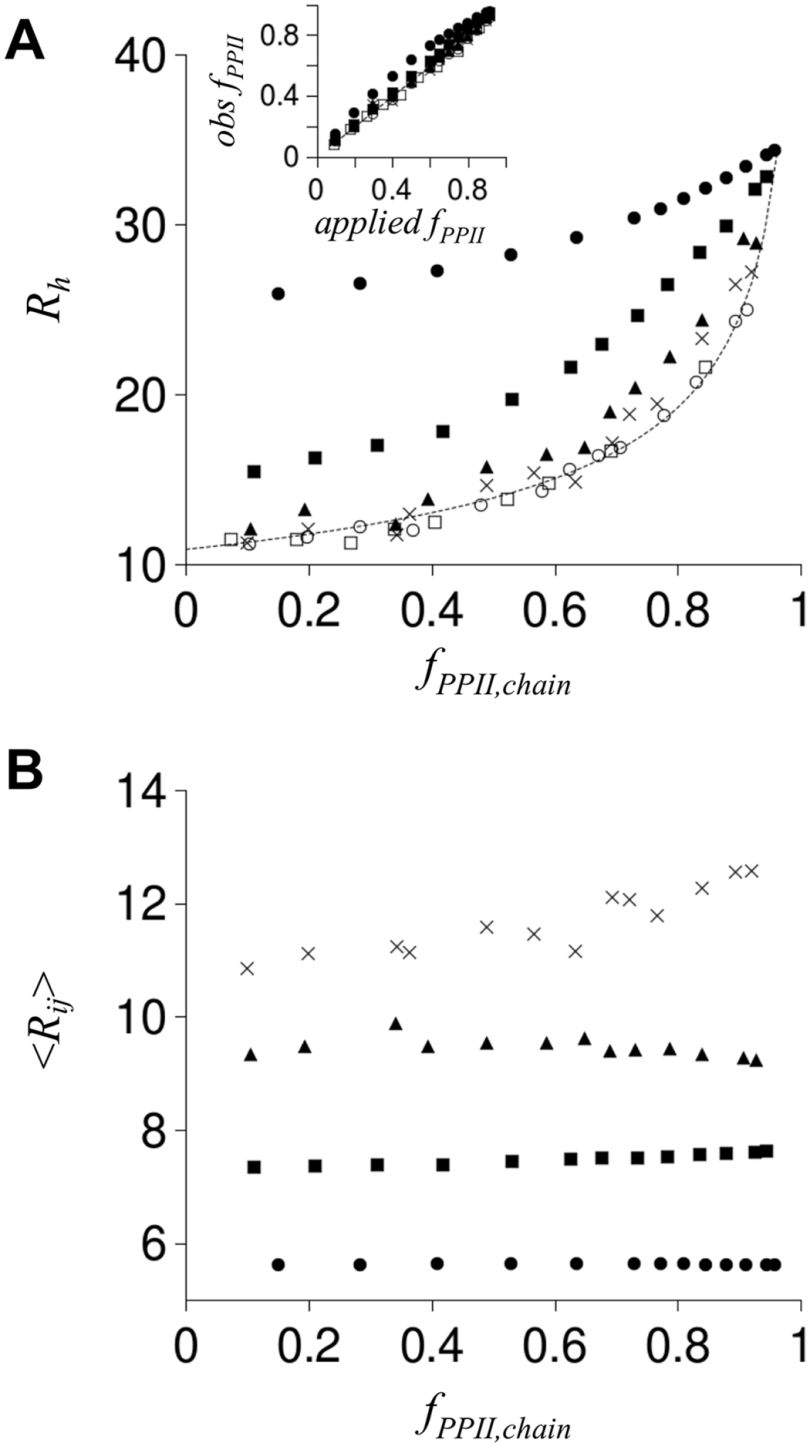
Simulated effect of charged residues on *R*_*h*_. In panel **A**, the stippled line is *R*_*h*_ from Eq (6) with *N* = 25 and *f*_*PPII*,*chain*_ = 0–0.98. Plotted symbols are *R*_*h*_ from poly-ALA simulations (*N* = 25) calculated using Eq (3). Open squares are uncharged poly-ALA and open circles have charged termini. Filled circles have each residue modeled with negative charge at the Cβ atom. Filled squares have every other residue modeled with negative charge, filled triangles have every third residue with negative charge, and X is every fourth residue with negative charge. In panel **B**, <*R*_*ij*_> is the ensemble averaged distance (in Å) between Cβ atoms from two charged residues, *i* and *j*, closest in sequence. Panel B symbols match panel A representations. **A inset:** comparison of observed *f*_*PPII*,*chain*_ (shown as *obs f*_*PPII*_) to *f*_*PPII*,*chain*_ expected from the applied *S*_*PPII*_ (shown as *applied f*_*PPII*_; calculated as *f*_*PPII*_ = *S*_*PPII*_ *−* 0.062∙exp(-(*S*_*PPII*_-0.63)^2^/(2∙0.28^2^)) [22]. Note that filled circles trend higher than other plotted data. Inset symbols match panel representations.

Fig 6A shows that introducing charge at N- and C-termini had no effect on simulated *R*_*h*_ for poly-ALA chains. Modeling negative charge at the Cβ position of each residue, or positive charge (S3 Fig), caused large increases in *R*_*h*_ from repulsive electrostatic intramolecular interactions. Identical charge at every other residue position caused smaller increases in *R*_*h*_, while identical charge at every third position gave *R*_*h*_ that were mostly similar to *R*_*h*_ of poly-ALA modeled with no charges. These data predict that the effects of charge on IDP *R*_*h*_ should weaken as charged residues separate in sequence, as expected. Fig 6B shows the ensemble-averaged distance between “charged” Cβ atoms that were closest in sequence for each ensemble in panel A, indicating repulsive charge-charge interactions at distances ≥9 Å had only minor effects on *R*_*h*_. The Debye length for the modeled conditions (i.e., 1/κ) was 9.6 Å, which is the distance where interactions between charged groups become negligible at a given ionic strength. The simulation results thus trend with expected outcomes for fully solvated charges. It was also observed that, for polypeptides with each residue position charged, *f*_*PPII*,*chain*_ calculated for an ensemble was larger than expected based upon the applied *S*_*PPII*_ (Fig 6A inset). This result predicts that repulsive charge-charge interactions between side chain groups preferentially select for the extended *PP*_*II*_ structure to minimize unfavorable interaction energies.

**Fig 7:**
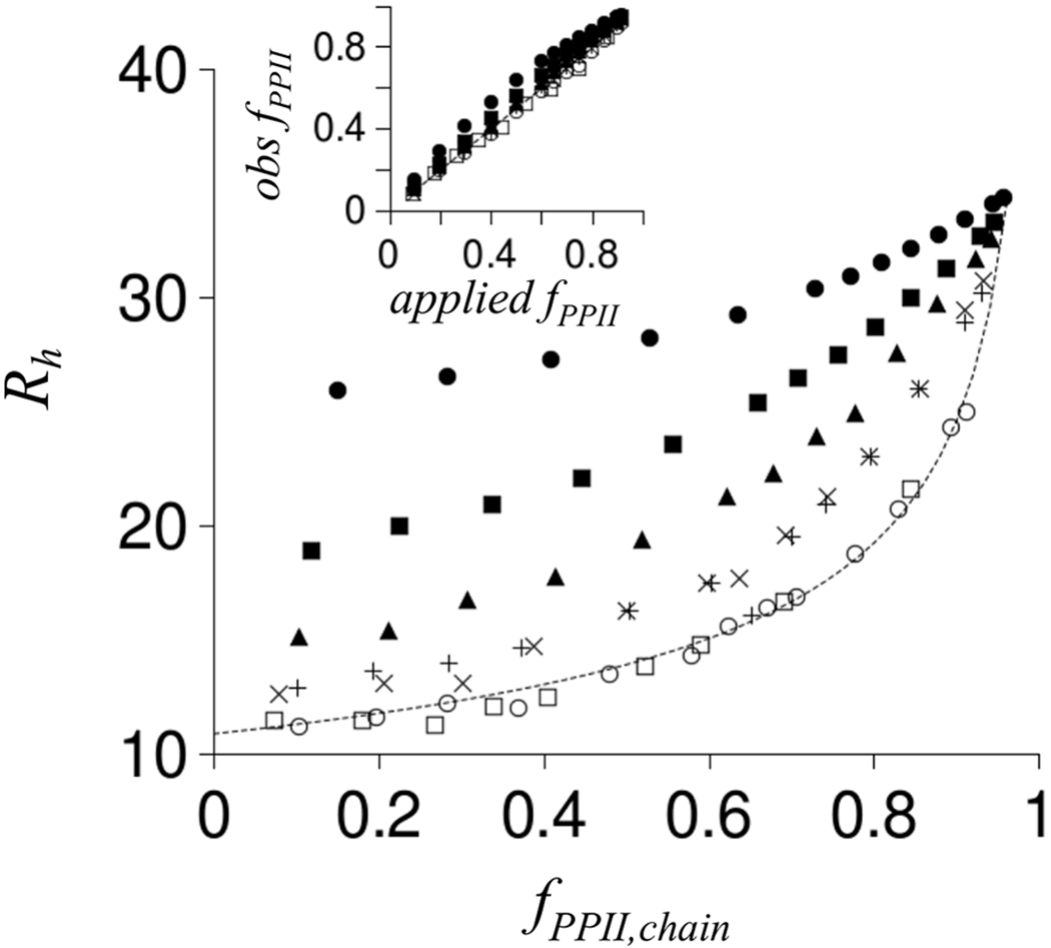
Simulated effect of clusters of charged residues on *R*_*h*_. Filled circles, open circles, open squares, and the stippled line were reproduced from Fig 6A. As in Fig 6A, *R*_*h*_ was calculated from poly-ALA simulations with *N* = 25. A charge cluster was defined as three consecutive residues with negative charge modeled at the Cβ atoms. Charge clusters separated in sequence by two uncharged residues (no charge modeled at Cβ) are shown with filled squares whereas charge clusters separated by four uncharged residues are shown with filled triangles. X and + symbols represent charge clusters separated by six and eight uncharged residues, respectively. **Inset:** comparison of observed *f*_*PPII*,*chain*_ to *f*_*PPII*,*chain*_ expected from the applied *S*_*PPII*_ (following Fig 6A inset description). Inset symbols match panel representations.

To test the effects of clusters of charge on *R*_*h*_, polypeptides with patterns consisting of three consecutively charged residues were also simulated (Fig 7). Similar trends were observed, whereby the effects of charge on *R*_*h*_ weaken as charged groups (i.e., clusters) were separated in sequence. Charge clusters, however, affected *R*_*h*_ when modeled with 4 intervening non-charged residues, with weaker effects persisting at even larger separation distances between the clusters. This contrasts with the simulation results for non-clustered charged residues that exhibited negligible effects on *R*_*h*_ when charges were separated by as little as 2 intervening uncharged residue positions (Fig 6A).

**Fig 8:**
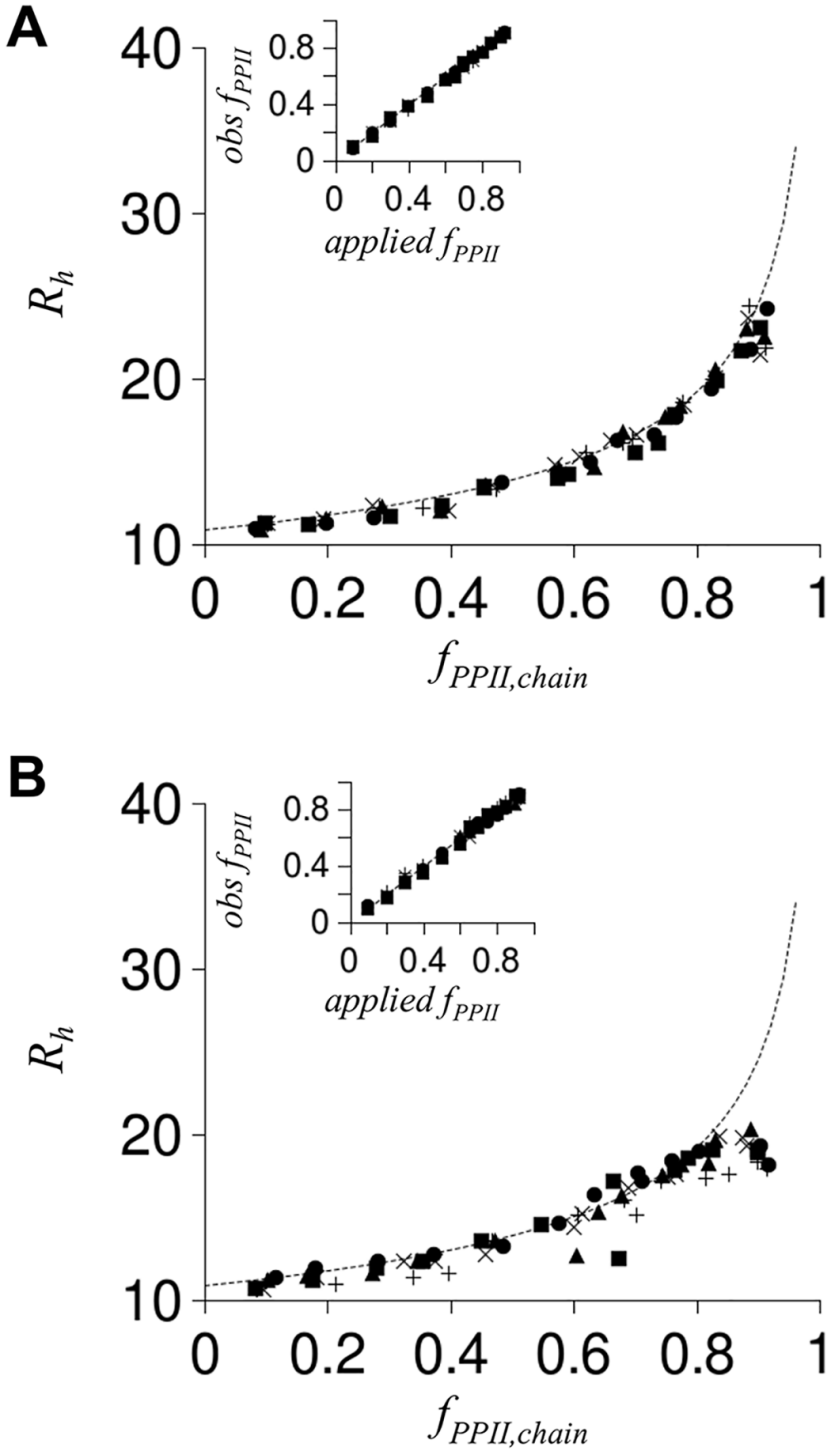
Simulated effect on *R*_*h*_ from oppositely charged residues. Stippled line in each panel was reproduced from Fig 6A. As in Fig 6A, *R*_*h*_ was calculated from poly-ALA simulations with *N* = 25. Charge was modeled with opposite charge at adjacent residue positions (panel A) or adjacent clusters (panel B). In panel **A**, filled circles have each residue modeled with charge at the Cβ atom (first residue negative, second residue positive, third residue negative, etc.). Filled squares have every other residue modeled with charge (first residue negative, third residue positive, etc.), filled triangles have every third residue modeled with charge, and X represents every fourth residue modeled with charge. In panel **B**, each residue in a cluster had identical charge while clusters adjacent in sequence had opposite charge. Filled circles are poly-ALA with every residue charged (i.e., residues 1–3 having negative charge, residues 4–6 with positive charge, residues 7–9 with negative charge, etc.). Charge clusters separated in sequence by two uncharged residues are shown with filled squares (i.e., residue 1–3 with negative charge, residues 4–5 uncharged, residues 6–8 with positive charge, etc.) whereas charge clusters separated by four uncharged residues are shown by filled triangles. X and + symbols represent charge clusters separated by six and eight uncharged residues, respectively. **Insets:** comparison of observed *f*_*PPII*,*chain*_ to *f*_*PPII*,*chain*_ expected from the applied *S*_*PPII*_ (following Fig 6A inset description). Inset symbols match panel representations.

Since IDPs, in general, contain both positive and negative charges, simulations with opposite charge at adjacent residue positions were also performed. Fig 8A shows that repeating patterns of opposite charge had minimal effects on *R*_*h*_ in these simulations, even when each residue position was charged. This was mostly the case for charge clusters too (Fig 8B) with the exception that the simulation would sporadically generate ensembles with compacted *R*_*h*_, whereby “compacted” is used to indicate *R*_*h*_ smaller than what was observed for non-charged poly-ALA coils of identical *N*. Overall, the amount of *R*_*h*_ compaction owing to favorable interactions between oppositely charged residues (or clusters) was small when compared to increases in *R*_*h*_ that were observed owing to unfavorable interactions between identically charged residues (or clusters).

**Fig 9:**
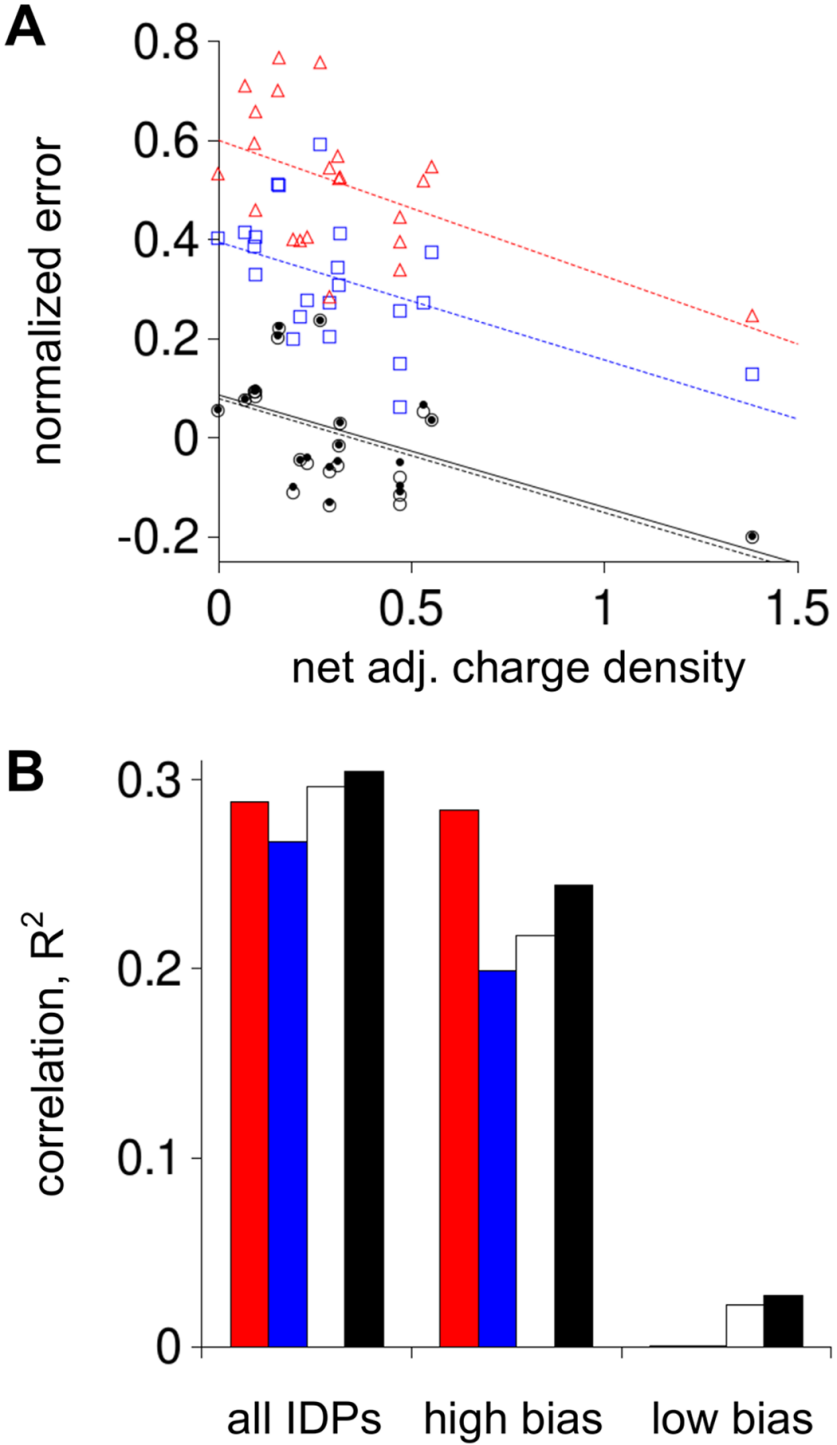
Correlation of normalized error in predicted *R*_*h*_ to net adjacent charge density. Panel **A** symbols and lines match their Fig 4 representations. Panel **B** shows correlations (R^2^) between normalized error and net adjacent charge density for all IDPs, IDPs in the high charge bias group (labeled as “high bias”), and IDPs in the low charge bias group (labeled as “low bias”). Red columns are correlations from using the Kallenbach propensity scale to predict *R*_*h*_, blue from using the Creamer propensities, white the Hilser propensities, and black the composite propensity scale.

The results in Figs 6–8 from modeling charge effects on *R*_*h*_ indicate that, in general, the strongest effects on *R*_*h*_ should occur owing to identical charges at sequentially-adjacent residue positions (Figs 6 and 7) and for polypeptides with the least amount of mixing of positive and negative charge types (Fig 8). To test these two general observations, the IDP dataset was analyzed to determine the net number of adjacent charges in each IDP sequence. This was calculated by first summing the number of ASP residues that had GLU or ASP immediately next or prior in sequence with the number of GLU residues that had GLU or ASP immediately next or prior in sequence to determine the total number of negative charges with an adjacent negatively charged neighbor. A similar calculation was performed using LYS and ARG to determine the number of positive charges with an adjacent positively charged neighbor. The net number of adjacent charges for an IDP was then the absolute value in the difference between the positive and negative adjacent charge numbers (provided in S1 Table). Fig 9A shows that normalized error in predicted *R*_*h*_ for the IDP dataset trends with the net adjacent charge density (i.e., net adjacent charge normalized for peptide length), similar to the correlation that was observed between normalized error and net charge density (Fig 4B). This should be expected since net charge and net adjacent charge correlate with R^2^ = 0.64 in the dataset.

The set of IDPs was also split according to the amount of mixing of positive and negative charge types in a given sequence. To do this, a “charge bias” was calculated for each IDP as the simple ratio of total negative charges (sum of ASP and GLU residues) to total positive charges (sum of LYS and ARG residues), or vice versa, depending on which ratio gave a value greater than 1. As a metric for separating IDPs with “high” and “low” charge bias, a “typical” charge bias was calculated for the entire dataset by the concatenated sequence and found to be 1.9. The average IDP charge bias, found to be 4.2, was not used to separate IDPs since: 1) ratio-based distributions are skewed, 2) only 7 IDPs would have been in the “high” charge bias set, and 3) 4 of these 7 were sequences derived from the p53 protein. Using the charge bias of the concatenated sequence gave 12 IDPs in the high charge bias set and 10 IDPs in the low charge bias set.

Fig 9B shows that correlations between net adjacent charge density and normalized error in predicted *R*_*h*_ persisted in the set of IDPs with high charge bias and mostly disappeared for IDPs with low charge bias, seeming to agree with the simulation prediction that significant mixing of positive and negative charge types in a sequence should reduce charge effects on *R*_*h*_. Applying this analysis to net charge density gave different results (S4 Fig). Correlations between net charge density and normalized error in predicted *R*_*h*_ decreased for both the high and low charge bias sets. This could be owing to trends shown in Fig 6, whereby net charge effects on *R*_*h*_ depended strongly on the distance between the charged groups. Overall, these results seem to indicate that charge effects on IDP structures are highly dependent on sequence, however, charge effects on *R*_*h*_ can be weakened substantially by mixing negative and positive charge types or by slight increases in the distances between charged groups in sequence. The hypothesis that charge effects on *R*_*h*_ may be generally weak for IDPs is supported by data in Fig 3B showing that *R*_*h*_ could be predicted without specific consideration of charges when provided an appropriate amino acid scale for intrinsic *PP*_*II*_ propensities.

## Discussion

Fig 1 shows that experimental *R*_*h*_ for IDPs are much larger than computational predictions based on random coil modeling of the *R*_*h*_ dependence on *N*. Numerous studies have demonstrated the importance of Coulombic effects for regulating IDP structural preferences [13–15]. Thus, it could be surprising to note that sequence effects on IDP *R*_*h*_ can be predicted with good agreement from sequence differences in *PP*_*II*_ propensity, even when other intramolecular factors are ignored. *R*_*h*_ predicted from IDP sequence and Eq (6) seemed to work best when using an experimental *PP*_*II*_ propensity scale from Hilser and colleagues [19], or a composite scale that combined the Hilser, Kallenbach [17], and Creamer [18] propensities, giving an average error of ~2.5 Å for an IDP dataset covering a wide range of residue lengths, net charge, and sequence composition. As examples of sequence differences in this dataset, the fractional number of PRO residues (*f*_*PRO*_ = (# PRO residues)/*N*) varied from 0 to 0.24, SER from 0.02 to 0.20, GLU from 0.06 to 0.31, and ALA from 0 to 0.16, indicating significant sequence diversity among the IDPs that were tested.

If it were established that molecular descriptions for *R*_*h*_ depend mostly on *PP*_*II*_ propensities for disordered proteins, this would have important implications. First, *R*_*h*_ well-above random coil estimates would indicate non-trivial preferences for *PP*_*II*_ structure. Fig 1 shows this to be the case for many IDPs. And second, large variations in *R*_*h*_ for IDPs with similar *N* would indicate large differences in propensity for *PP*_*II*_ structure among the biologically common amino acids. Observed differences in amino acid propensity for *PP*_*II*_ [17–19,53] are thus consistent with the observed differences in *R*_*h*_ for IDPs with similar *N*. For example, consider that *R*_*h*_ varied from 24.5 Å to 32.4 Å for IDPs with *N* = 87–97 in Fig 1. The average prediction error in *R*_*h*_ for these 8 IDPs from using Eq (6) and the composite propensity scale was only 1.7 ± 0.7 Å, though net charge ranged from 4 to 29 for these proteins. In contrast, predictions using random coil values give *R*_*h*_ from 20.5 to 21.7 Å with an average error of 6.4 ± 2.7 Å.

The simulation-derived relationship between *R*_*h*_, *N*, and *f*_*PPII*,*chain*_ appears to be surprisingly simple for disordered proteins. As noted above, Eq (6) should be interpreted as an ideal relationship that excludes many molecular factors known to regulate structural preferences in proteins (e.g., electrostatic effects, *cis*-*trans* isomerization rates). Observed deviations from this “ideal” behavior can then be interpreted in terms of factors that were not modeled, as shown (Fig 4B). We recognize that exclusive use of poly-ALA for computational modeling may prove to be unjustified with further studies. Poly-ALA was used as a simplifying step since the effects of *N* on *R*_*h*_ were mostly independent of amino acid sequence in previous HSC-based simulations and agreed with general IDP trends determined from a literature survey [22,49]. As shown here, this simulation-derived relationship provides a straight-forward molecular explanation for *R*_*h*_ variations among IDPs. The *R*_*h*_ dependence on *f*_*PPII*,*chain*_ also predicts heat-induced compaction of IDP *R*_*h*_ since the enthalpy of unfolding *PP*_*II*_ structure is positive [16,64]. Many studies have demonstrated *R*_*h*_ compaction caused by elevated temperatures for IDPs [22,43,44].

As noted above, the simulation results presented here could be interpreted as indicating that charge effects on *R*_*h*_ are generally weak for IDPs, relative to the effects of intrinsic *PP*_*II*_ propensities. These data demonstrate, however, that certain sequence patterns of charge can modulate *R*_*h*_ substantially (see Fig 6). For charged groups, this would be those that are separated at distances averaging less than the solution Debye length, involving identical charge type (i.e., positive or negative), and within a region showing higher than typical charge bias. These general rules are in qualitative agreement with results from Pappu and colleagues showing that simulated hydrodynamic sizes for highly charged and disordered polypeptides, with every residue modeled as GLU or LYS, depend strongly on the mixing of negative and positive charge types [15]. In that study, mixing of charge types in a sequence caused structural compaction relative to biased charge distributions, similar to our own conclusions. The observation that unfavorable charge-charge interactions between side chain groups can promote *PP*_*II*_ structure (Figs 6A and 7 insets) has also been noticed in computational studies from other researchers [14,65]. This result predicts multiple mechanisms for charge-mediated regulation of IDP structure; possibly owing to both the accumulation of charge and local modulation of *PP*_*II*_ propensities. Overall, these data demonstrate the importance of sequence context for understanding the structural properties of IDPs and for describing quantitatively how disordered protein structures respond to discrete perturbations such as changes in charge state and amino acid substitutions.

## Methods

### Computer generation of polypeptide structures

Detailed description of the computer algorithm that was used is provided elsewhere [22,24]. Briefly, simulations of disordered protein structures were limited to poly-ALA polypeptides. Main chain atoms of poly-ALA were generated using the standard bond angles and bond lengths [66] and a random sampling of the dihedral angles Φ, Ψ, and ω. The dihedral angle ω was given a Gaussian fluctuation of ±5° around the *trans* value of 180°. To sample conformational space efficiently, (Φ,Ψ) values were restricted to the allowed Ramachandran regions [67]. Of the two possible positions of the side chain Cβ atom, the one corresponding to L-alanine was used throughout the studies. To calculate state distributions typical of protein ensembles, a structure-based energy function parameterized to solvent-accessible surface areas was used to population-weight the generated structures [54–62].

## Supporting Information

S1 Fig Comparison of *f*_*PPII*_ and *S*_*PPII*_. In this figure, *S*_*PPII*_ is the average applied sampling rate for *PP*_*II*_ for residues with *S*_*PPII*_ ≠ 0 in a simulation, while *f*_*PPII*_ was the observed per-position average *PP*_*II*_ rate, also excluding residues with *S*_*PPII*_ = 0. Open circles are from ensembles where position-specific *S*_*PPII*_ followed the pattern specified in the text (i.e., different simulations had different *S*_*PPII*_ ranging from 0.1 to 0.9 in 0.1 increments applied to each residue, every other residue, every third residue, etc.) which is why circles align at *S*_*PPII*_ = 0.1–0.9 in 0.1 increments. Blue circles give the average *f*_*PPII*_ for each applied *S*_*PPII*_. Open squares represent this calculation performed on simulations using randomly assigned position-specific *S*_*PPII*_. Stippled line is the identity; solid line is the relationship between *f*_*PPII*_ and *S*_*PPII*_ established previously for *S*_*PPII*_ applied at constant values across all residues [22]. In general, *f*_*PPII*_ trends with *S*_*PPII*_ by: *f*_*PPII*_ = *S*_*PPII*_-0.062∙exp(-(*S*_*PPII*_-0.63)^2^/(2∙0.28^2^)). This gives the algorithm the ability to target specific *f*_*PPII*_ from the applied value of *S*_*PPII*_.
(TIF)

S2 Fig Correlation of experimental *PP*_*II*_ propensities for the common amino acids. Panel **A**, correlation of Kallenbach [17] and Creamer reported values [18]. Panel **B**, correlation of Kallenbach and Hilser reported values [19]. Panel **C**, correlation of Creamer and Hilser reported values. Panel **D**, correlation of Creamer and Zondlo reported values [53].
(TIF)

S3 Fig Simulated effect of positive charged residues on *R*_*h*_. Stippled line is *R*_*h*_ from Eq (6) with *N* = 25 and *f*_*PPII*,*chain*_ from 0 to 0.98. Symbols are simulated *R*_*h*_ from ensembles of poly-ALA (*N* = 25) using Eq (3) (*R*_*h*_ = <*L*>/2). Filled circles have each residue modeled with positive charge at the Cβ atom. Filled squares have every other residue modeled with positive charge, filled triangles have every third residue modeled with positive charge, and X represents every fourth residue modeled with positive charge. **Inset:** comparison of observed *f*_*PPII*,*chain*_ to *f*_*PPII*,*chain*_ expected from the applied *S*_*PPII*_ (following Fig 6A inset description). Inset symbols match panel representations.
(TIF)

S4 Fig Correlation of normalized error in predicted *R*_*h*_ to net charge density. Shown are correlations (R^2^) between normalized error and net charge density for all IDPs, IDPs in the high charge bias group (labeled as “high bias”), and IDPs in the low charge bias group (labeled as “low bias”). Red columns are correlations from using the Kallenbach propensity scale to predict *R*_*h*_, blue from using the Creamer propensities, white the Hilser propensities, and black the composite propensity scale.
(TIF)

S1 Table IDP dataset. (DOCX)

S2 Table Sequence of each IDP in dataset. (DOCX)
