# Supplementary figures and images for: Hydrodynamic Radii of Intrinsically Disordered Proteins Determined from Experimental Polyproline II Propensities
Source: PLoS Comput Biol. 2016 Jan 4;12(1):e1004686. doi: 10.1371/journal.pcbi.1004686 (PMC4699819; doi:10.1371/journal.pcbi.1004686)

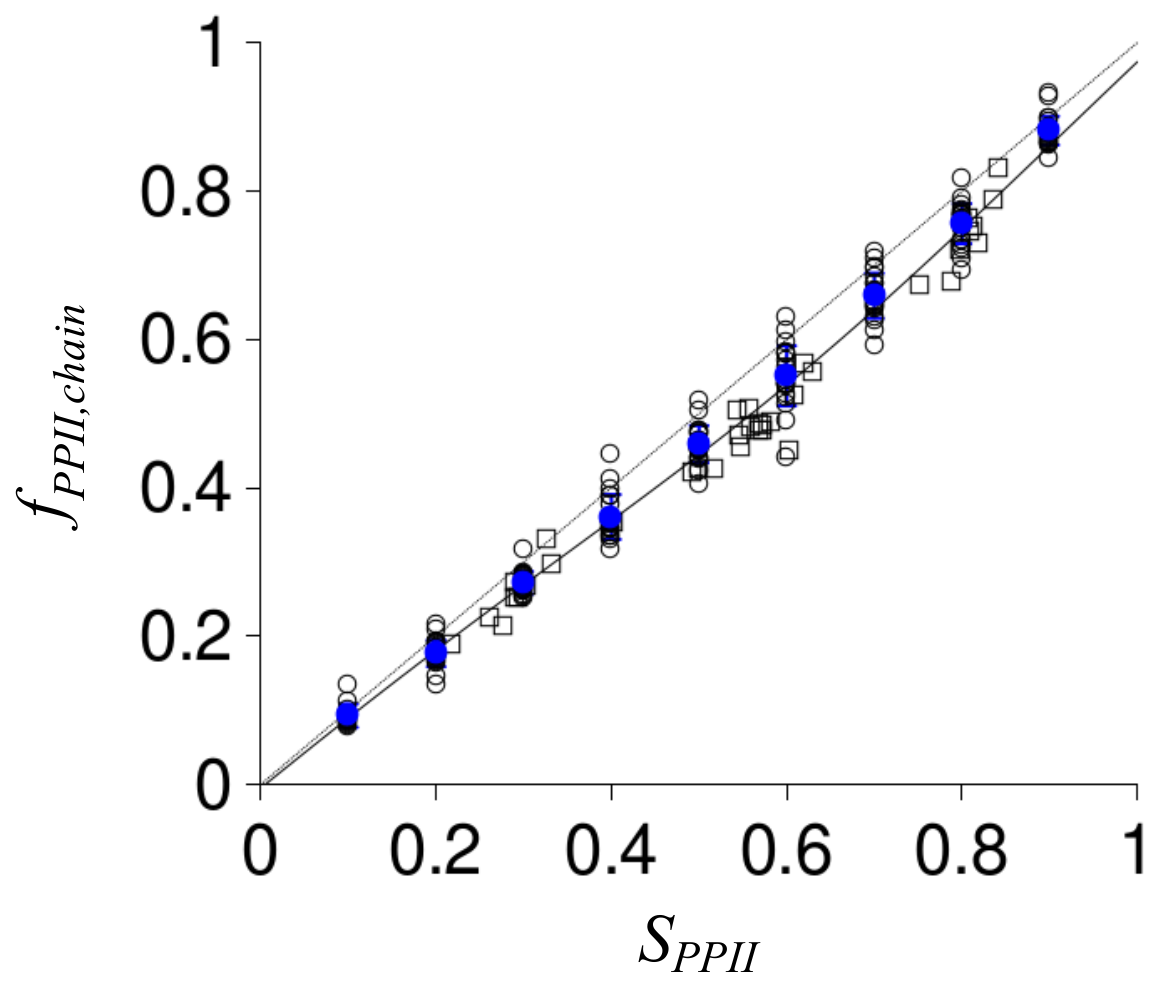

Supplement: S1 Fig — In this figure, S PPII is the average applied sampling rate for PP II for residues with S PPII ≠ 0 in a simulation, while f PPII was the observed per-position average PP II rate, also excluding residues with S PPII = 0. Open circles are from ensembles where position-specific S PPII followed the pattern specified in the text (i.e., different simulations had different S PPII ranging from 0.1 to 0.9 in 0.1 increments applied to each residue, every other residue, every third residue, etc.) which is why circles align at S PPII = 0.1–0.9 in 0.1 increments. Blue circles give the average f PPII for each applied S PPII. Open squares represent this calculation performed on simulations using randomly assigned position-specific S PPII. Stippled line is the identity; solid line is the relationship between f PPII and S PPII established previously for S PPII applied at constant values across all residues [22]. In general, f PPII trends with S PPII by: f PPII = S PPII-0.062∙exp(-(S PPII-0.63)2/(2∙0.282)). This gives the algorithm the ability to target specific f PPII from the applied value of S PPII. (TIF) [file pcbi.1004686.s001.tif]

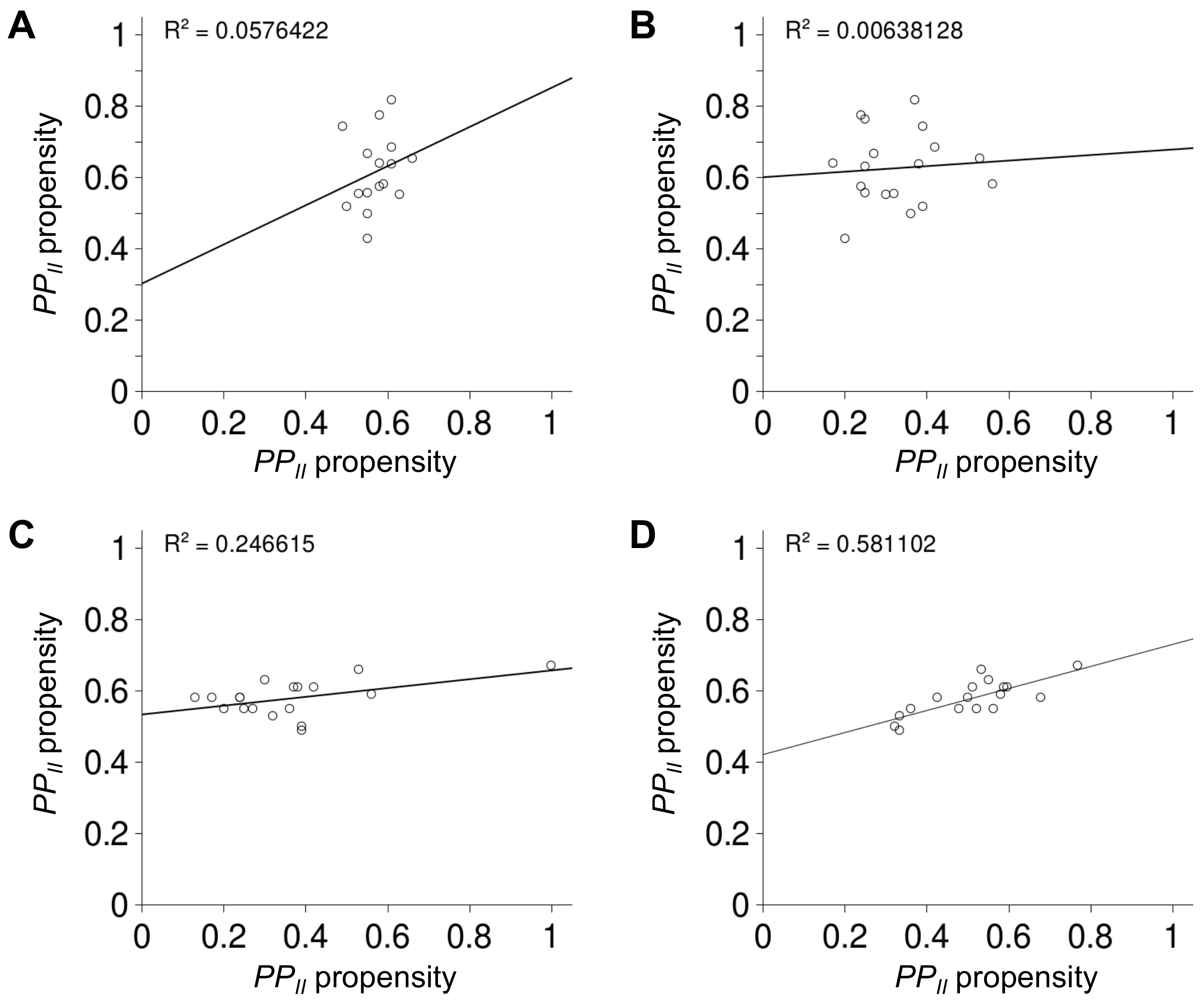

Supplement: S2 Fig — Panel A, correlation of Kallenbach [17] and Creamer reported values [18]. Panel B, correlation of Kallenbach and Hilser reported values [19]. Panel C, correlation of Creamer and Hilser reported values. Panel D, correlation of Creamer and Zondlo reported values [53]. (TIF) [file pcbi.1004686.s002.tif]

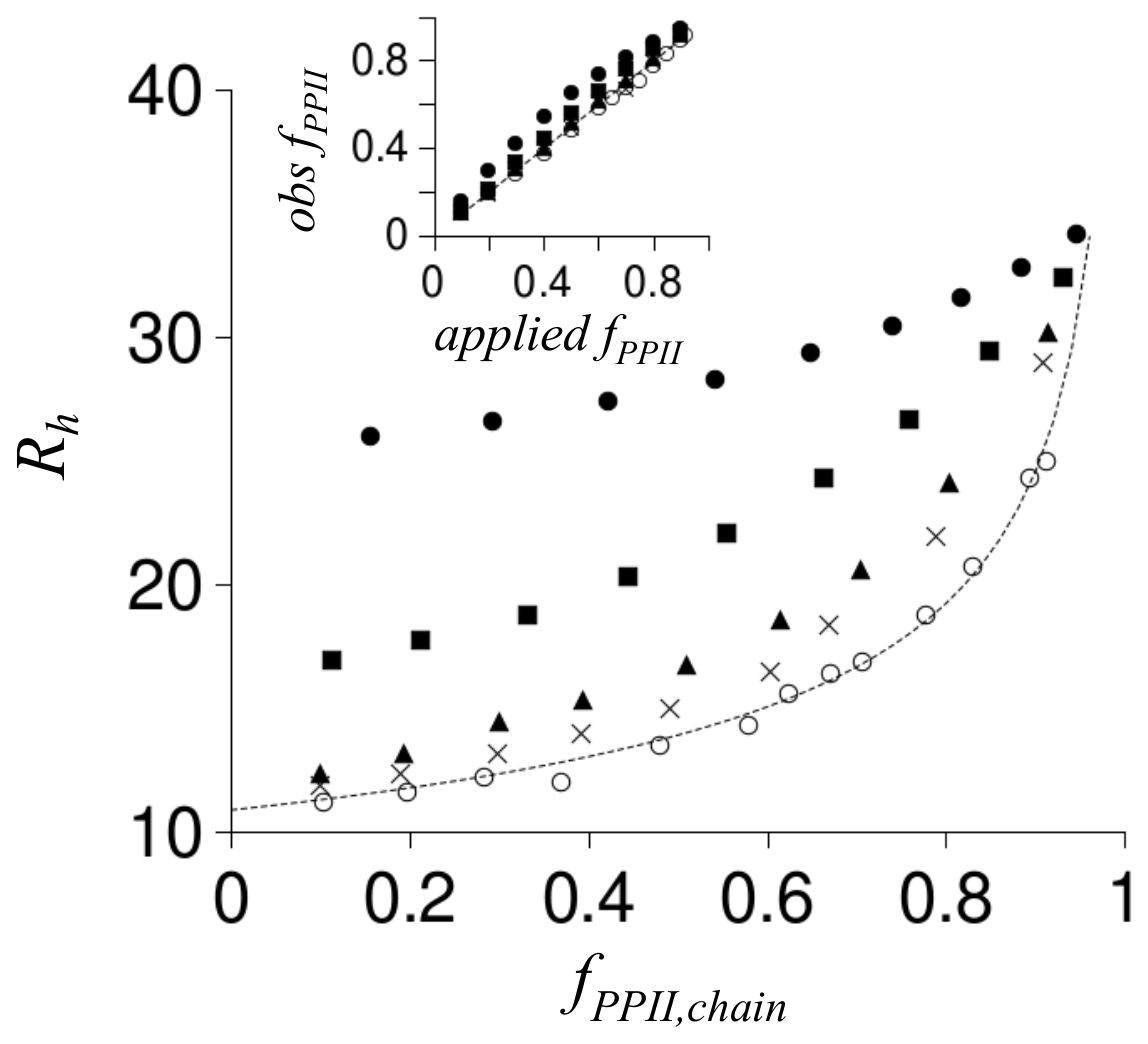

Supplement: S3 Fig — Stippled line is R h from Eq (6) with N = 25 and f PPII,chain from 0 to 0.98. Symbols are simulated R h from ensembles of poly-ALA (N = 25) using Eq (3) (R h = /2). Filled circles have each residue modeled with positive charge at the Cβ atom. Filled squares have every other residue modeled with positive charge, filled triangles have every third residue modeled with positive charge, and X represents every fourth residue modeled with positive charge. Inset: comparison of observed f PPII,chain to f PPII,chain expected from the applied S PPII (following Fig 6A inset description). Inset symbols match panel representations. (TIF) [file pcbi.1004686.s003.tif]

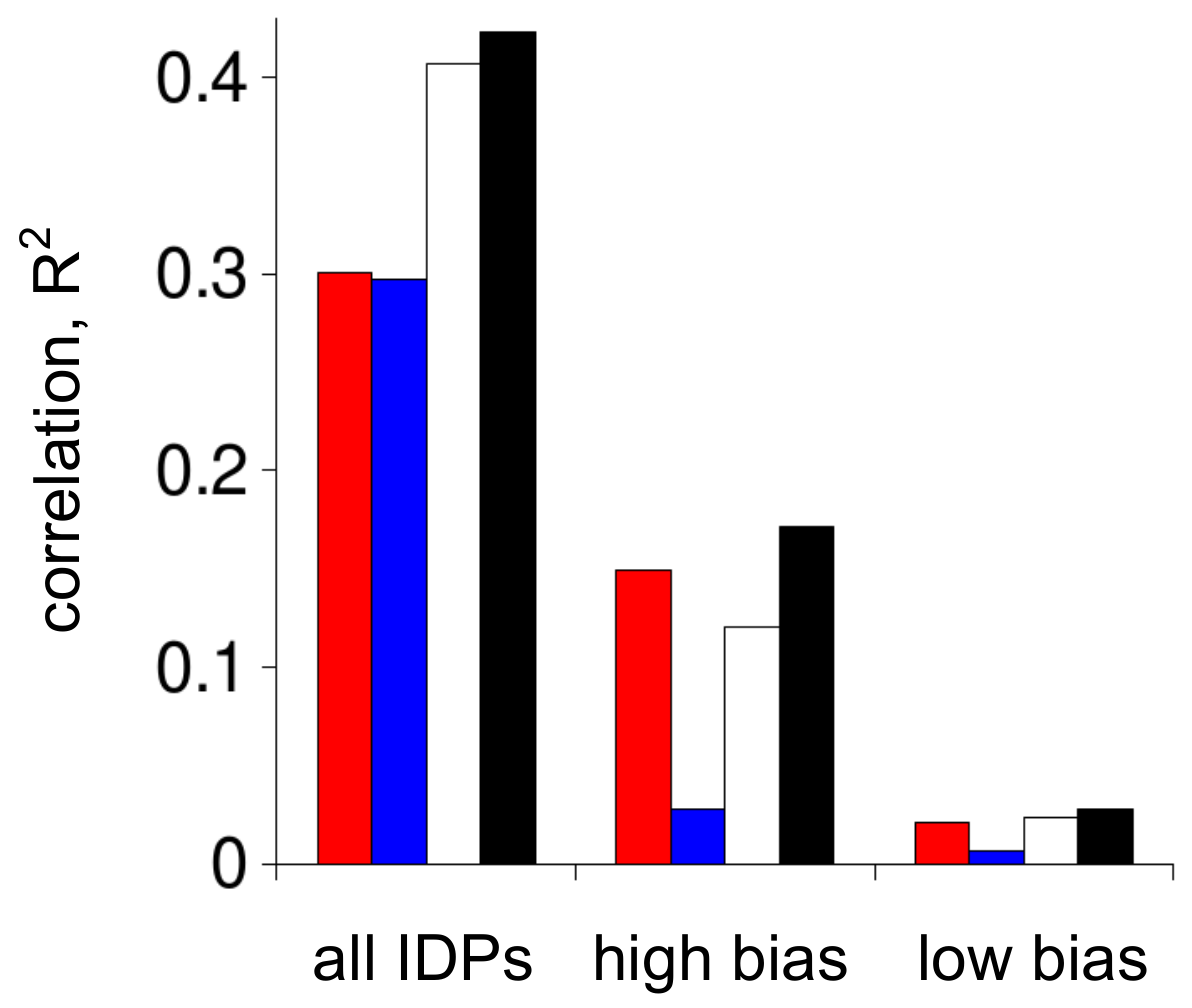

Supplement: S4 Fig — Shown are correlations (R2) between normalized error and net charge density for all IDPs, IDPs in the high charge bias group (labeled as “high bias”), and IDPs in the low charge bias group (labeled as “low bias”). Red columns are correlations from using the Kallenbach propensity scale to predict R h, blue from using the Creamer propensities, white the Hilser propensities, and black the composite propensity scale. (TIF) [file pcbi.1004686.s004.tif]
