# Supplementary material for: Hydrodynamic Radii of Intrinsically Disordered Proteins Determined from Experimental Polyproline II Propensities
Source: PLoS Comput Biol. 2016 Jan 4;12(1):e1004686. doi: 10.1371/journal.pcbi.1004686 (PMC4699819; doi:10.1371/journal.pcbi.1004686)
Supplement: S1 Table — (DOCX) [file pcbi.1004686.s005.docx]

**S1 Table. IDP dataset.**

| IDP | *N* | *R_h_*^a^ | net charge | # D E^b^ | # K R^c^ | net adj charge | neg adj charge^d^ | pos adj charge^e^ | charge bias |
| --- | --- | --- | --- | --- | --- | --- | --- | --- | --- |
| p53(1-93) | 93 | 32.4 | 15 | 17 | 2 | 10 | 10 | 0 | 8.50 |
| p53(1-93) ALA- | 93 | 30.4 | 15 | 17 | 2 | 10 | 10 | 0 | 8.50 |
| p53(1-93) PRO- | 93 | 27.4 | 15 | 17 | 2 | 10 | 10 | 0 | 8.50 |
| p53 TAD | 73 | 23.8 | 14 | 17 | 3 | 10 | 10 | 0 | 5.67 |
| Vmw65 | 89 | 28 | 19 | 21 | 2 | 6 | 8 | 2 | 10.50 |
| Hdm2-ABD | 97 | 25.7 | 29 | 31 | 2 | 12 | 12 | 0 | 15.50 |
| prothymosin-α | 110 | 33.7 | 43 | 53 | 10 | 32 | 38 | 6 | 5.30 |
| HIF1-α-403 | 202 | 44.3 | 29 | 38 | 9 | 9 | 9 | 0 | 4.22 |
| Fos-AD | 168 | 35 | 16 | 23 | 7 | 2 | 4 | 2 | 3.29 |
| Mlph(147-240) | 97 | 28 | 15 | 23 | 8 | 5 | 10 | 5 | 2.88 |
| Tau-K45 | 198 | 45 | 19 | 15 | 34 | 6 | 0 | 6 | 2.27 |
| Mlph(147-403) | 260 | 49 | 28 | 51 | 23 | 11 | 24 | 13 | 2.22 |
| p57-ID | 73 | 24 | 6 | 14 | 8 | 4 | 4 | 0 | 1.75 |
| PDE-γ | 87 | 24.8 | 4 | 9 | 13 | 2 | 2 | 4 | 1.44 |
| LJIDP1 | 94 | 24.52 | 4 | 10 | 14 | 2 | 4 | 2 | 1.40 |
| Cad136 | 136 | 28.1 | 9 | 14 | 23 | 4 | 0 | 4 | 1.64 |
| α-synuclein | 140 | 28.2 | 9 | 24 | 15 | 4 | 6 | 2 | 1.60 |
| CFTR-R-region | 189 | 32 | 5 | 30 | 25 | 8 | 12 | 20 | 1.20 |
| SNAP25 | 206 | 39.7 | 14 | 43 | 29 | 10 | 18 | 8 | 1.48 |
| ShB-C | 146 | 32.9 | 4 | 10 | 6 | 0 | 2 | 2 | 1.67 |
| HIF1-α-530 | 170 | 38.3 | 10 | 26 | 16 | 9 | 9 | 0 | 1.63 |
| Securin | 202 | 39.7 | 1 | 27 | 26 | 3 | 7 | 4 | 1.04 |

*^a^*reported in Å, reproduced from [23,26-42]

*^b^*number of ASP (D) and GLU (E) residues in IDP sequence (S2 Table)

*^c^*number of LYS (K) and ARG (R) residues in IDP sequence (S2 Table)

*^d^*number of negative charge residues (D and E) adjacent in sequence to another negative charge residue

*^e^*number of positive charge residues (K and R) adjacent in sequence to another positive charge residue
