# Supplementary material for: Hydrodynamic Radii of Intrinsically Disordered Proteins Determined from Experimental Polyproline II Propensities
Source: PLoS Comput Biol. 2016 Jan 4;12(1):e1004686. doi: 10.1371/journal.pcbi.1004686 (PMC4699819; doi:10.1371/journal.pcbi.1004686)
Supplement: S2 Table — (DOCX) [file pcbi.1004686.s006.docx]

**S2 Table. Sequence of each IDP in dataset.**

| IDP | Sequence |
| --- | --- |
| p53(1-93) | MEEPQSDPSVEPPLSQETFSDLWKLLPENNVLSPLPSQAMDDLMLSPDDIEQWFTEDPGPDEAPRMPEAAPPVAPAPAAPTPAAPAPAPSWPL |
| p53(1-93) ALA- | MEEPQSDPSVEPPLSQETFSDLWKLLPENNVLSPLPSQGMDDLMLSPDDIEQWFTEDPGPDEGPRMPEGGPPVGPGPGGPTPGGPGPGPSWPL |
| p53(1-93) PRO- | MEEGQSDGSVEGGLSQETFSDLWKLLGENNVLSGLGSQAMDDLMLSGDDIEQWFTEDGGGDEAGRMGEAAGGVAGAGAAGTGAAGAGAGSWGL |
| p53 TAD | MEEPQSDPSVEPPLSQETFSDLWKLLPENNVLSPLPSQAMDDLMLSPDDIEQWFTEDPGPDEAPRMPEAAPRV |
| Vmw65 | GSAGHTRRLSTAPPTDVSLGDELHLDGEDVAMAHADALDDFDLDMLGDGDSPGPGFTPHDSAPYGALDMADFEFEQMFTDALGIDEYGG |
| Hdm2-ABD | ERSSSSESTGTPSNPDLDAGVSEHSGDWLDQDSVSDQFSVEFEVESLDSEDYSLSEEGQELSDEDDEVYQVTVYQAGESDTDSFEEDPEISLADYWK |
| prothymosin-α | MSDAAVDTSSEITTKDLKEKKEVVEEAENGRDAPANGNANEENGEQEADNEVDEEEEEGGEEEEEEEEGDGEEEDGDEDEEAESATGKRAAEDDEDDDVDTKKQKTDEDD |
| HIF1-α-403 | PAAGDTIISLDFGSNDTETDDQQLEEVPLYNDVMLPSPNEKLQNINLAMSPLPTAETPKPLRSSADPALNQEVALKLEPNPESLELSFTMPQIQDQTPSPSDGSTRQSSPEPNSPSEYCFYVDSDMVNEFKLELVEKLFAEDTEAKNPFSTQDTDLDLEMLAPYIPMDDDFQLRSFDQLSPLESSSASPESASPQSTVTVFQ |
| Fos-AD | GSHMSVASLDLTGGLPEVATPESEEAFTLPLLNDPEPKPSVEPVKSISSMELKTEPFDDFLFPASSRPSGSETARSVPDMDLSGSFYAADWEPLHSGSLGMGPMATELEPLCTPVVTCTPSCTAYTSSFVFTYPEADSFPSCAAAHRKGSSSNEPSSDSLSSPTLLAL |
| Mlph(147-240) | RLQGGGGSEPSLEEGNGDSEQTDEDGDLDTEARDQPLNSKKKKRLLSFRDVDFEEDSDHLVQPCSQTLGLSSVPESAHSLQSLSGEPYSEDTTSLEP |
| Tau-K45 | MSSPGSPGTPGSRSRTPSLPTPPTREPKKVAVVRTPPKSPSSAKSRLQTAPVPMPDLKNVKSKIGSTENLKHQPGGGKVQIINKKLDLSNVQSKCGSKDNIKHVPGGGSVQIVYKPVDLSKVTSKCGSLGNIHHKPGGGQVEVKSEKLDFKDRVQSKIGSLDNITHVPGGGNKKIETHKLTFRENAKAKTDHGAEIVY |
| Mlph(147-403) | RLQGGGGSEPSLEEGNGDSEQTDEDGDLDTEARDQPLNSKKKKRLLSFRDVDFEEDSDHLVQPCSQTLGLSSVPESAHSLQSLSGEPYSEDTTSLEPEGLEETGARALGCRPSPEVQPCSPLPSGEDAHAELDSPAASCKSAFGTTAMPGTDDVRGKHLPSQYLADVDTSDEDSIQGPRAASQHSKRRARTVPETQILELNKRMSAVEHLLVHLENTVLPPSAQEPTVETHPSADTEEETLRRRLEELTSNISGSSTSSE |
| p57-ID | VRTSACRSLFGPVDHEELSRELQARLAELNAEDQNRWDYDFQQDMPLRGPGRLQWTEVDSDSVPAFYRETVQV |
| PDE-γ | MNLEPPKAEIRSATRVMGGPVTPRKGPPKFKQRQTRQFKSKPPKKGVQGFGDDIPGMEGLGTDITVICPWEAFNHLELHELAQYGII |
| LJIDP1 | MARSFTNIKAISALVAEEFSNSLARRGYAATAQSAGRVGASMSGKMGSTKSGEEKAAAREKVSWVPDPVTGYYKPENIKEIDVAELRSAVLGKN |
| Cad136 | RLEQYTSAVVGNKAAKPAKPAASDLPVPAEGVRNIKSMWEKGNVFSSPGGTGTPNKETAGLKVGVSSRINEWLTKTPEGNKSPAPKPSDLRPGDVSGKRNLWEKQSVEKPAASSSKVTATGKKSETNGLRQFEKEP |
| α-synuclein | MDVFMKGLSKAKEGVVAAAEKTKQGVAEAAGKTKEGVLYVGSKTKEGVVHGVATVAEKTKEQVTNVGGAVVTGVTAVAQKTVEGAGSIAAATGFVKKDQLGKNEEGAPQEGILEDMPVDPDNEAYEMPSEEGYQDYEPEA |
| CFTR-R-region | GAMESAERRNSILTETLHRFSLEGDAPVSWTETKKQSFKQTGEFGEKRKNSILNPINSIRKFSIVQKTPLQMNGIEEDSDEPLERRLSLVPDSEQGEAILPRISVISTGPTLQARRRQSVLNLMTHSVNQGQNIHRKTTASTRKVSLAPQANLTELDIYSRRLSQETGLEISEEINEEDLKECLFDDME |
| SNAP25 | MAEDADMRNELEEMQRRADQLADESLESTRRMLQLVEESKDAGIRTLVMLDEQGEQLERIEEGMDQINKDMKEAEKNLTDLGKFCGLCVCPCNKLKSSDAYKKAWGNNQDGVVASQPARVVDEREQMAISGGFIRRVTNDARENEMDENLEQVSGIIGNLRHMALDMGNEIDTQNRQIDRIMEKADSNKTRIDEANQRATKMLGSG |
| ShB-C | MTLGQHMKKSSLSESSSDMMDLDDGVESTPGLTETHPGRSAVAPFLGAQQQQQQPVASSLSMSIDKQLQHPLQQLTQTQLYQQQQQQQQQQQNGFKQQQQQTQQQLQQQQSHTINASAAAATSGSGSSGLTMRHNNALAVSIETDV |
| HIF1-α-530 | NEFKLELVEKLFAEDTEAKNPFSTQDTDLDLEMLAPYIPMDDDFQLRSFDQLSPLESSSASPESASPQSTVTVFQQTQIQEPTANATTTTATTDELKTVTKDRMEDIKILIASPSPTHIHKETTSATSSPYRDTQSRTASPNRAGKGVIEQTEKSHPRSPNVLSVALSQR |
| Securin | MATLIYVDKENGEPGTRVVAKDGLKLGSGPSIKALDGRSQVSTPRFGKTFDAPPALPKATRKALGTVNRATEKSVKTKGPLKQKQPSFSAKKMTEKTVKAKSSVPASDDAYPEIEKFFPFNPLDFESFDLPEEHQIAHLPLSGVPLMILDEERELEKLFQLGPPSPVKMPSPPWESNLLQSPSSILSTLDVELPPVCCDIDI |
